# Correlation of the differential expression of *PIK3R1* and its spliced variant, p55α, in pan‐cancer

**DOI:** 10.1002/1878-0261.70205

**Published:** 2026-01-20

**Authors:** Ishita Gupta, Yang Song, Madeleine Ndahayo, Anirudh Saxena, Theresa Guo, Dylan Z. Kelley, Jessica Gore, Andrew Hennigan, Alexa Anderson, John C. Papadimitriou, Daria A. Gaykalova

**Affiliations:** ^1^ Institute for Genome Sciences University of Maryland School of Medicine Baltimore MD USA; ^2^ Department of Otorhinolaryngology‐Head and Neck Surgery, Marlene & Stewart Greenebaum Comprehensive Cancer Center University of Maryland Medical Center Baltimore MD USA; ^3^ Department of Oncology, Sidney Kimmel Comprehensive Cancer Center Johns Hopkins University Baltimore MD USA; ^4^ Department of Pathology University of Maryland School of Medicine, University of Maryland Medical Center Baltimore MD USA

**Keywords:** cancer, p55α, p85α, *PIK3R1*, race, splicing variant

## Abstract

*PIK3R1*, a regulatory subunit of class IA phosphoinositide‐3‐kinase (PI3K), undergoes alternative splicing to generate multiple isoforms, primarily p85α and p55α. The canonical isoform p85α associates with the catalytic subunit p110α to form the active PI3K complex, which regulates key cellular functions such as growth, proliferation, survival, and metabolism. In this study, we performed a comprehensive pan‐cancer analysis integrating transcriptomic, proteomic, and genomic data to investigate the expression patterns of p85α and its splicing variant, p55α, and their associations with clinical outcomes. Our findings reveal that while p85α expression is significantly reduced, p55α is elevated in tumors as compared to normal samples. These alterations are linked to poor prognosis across multiple cancer types. Notably, we observed racial disparities in expression patterns, with African American patients exhibiting more pronounced downregulation of p85α and upregulation of p55α than European Americans, potentially contributing to differential clinical outcomes. This is the first study to systematically evaluate p85α and p55α expression across diverse cancers and populations, highlighting the role of alternative splicing in PI3K pathway dysregulation and its relevance to cancer progression and health disparities.

## Graphical abstract

*PIK3R1* undergoes alternative splicing to generate the isoforms, p85α and p55α. By combining large patient datasets with laboratory experiments, we show that *PIK3R1* spliced variants shape cancer behavior. While tumors lose the protective p85α isoform, p55α is overexpressed, changes linked to poorer survival and more pronounced in African American patients, highlighting a potential driver of cancer progression and health disparities.

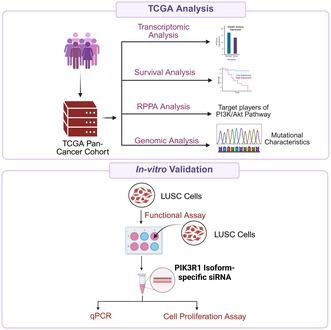

**Abbreviations**
AA: African American
ACC: adrenocortical carcinoma
Akt: Ak strain transforming
ASE: alternative splicing event
BLCA: Bladder Urothelial carcinoma
BRCA: breast invasive carcinoma
CESC: cervical squamous cell carcinoma and endocervical adenocarcinoma
CHOL: cholangiocarcinoma
DLBC: Lymphoid Neoplasm Diffuse Large B‐cell Lymphoma
EA: European American
ESCA: esophageal carcinoma
GBM: glioblastoma multiforme
GDC: Genomic Data Commons
gnomAD: Genome Aggregation Database
HNSC: head and neck squamous cell carcinoma
IRS: insulin receptor substrate
KICH: kidney chromophobe
KIRC: kidney renal clear cell carcinoma
KIRP: kidney renal papillary cell carcinoma
LAML: acute myeloid leukemia
LGG: Brain Lower Grade Glioma
LIHC: liver hepatocellular carcinoma
LUAD: lung adenocarcinoma
LUSC: lung squamous cell carcinoma
MESO: mesothelioma
miRNA: microRNA
mTOR: Mammalian Target of Rapamycin
mTORC: Mammalian Target of Rapamycin Complex
NHBE: normal bronchial/tracheal epithelial
OS: overall survival
OV: ovarian serous cystadenocarcinoma
PAAD: pancreatic adenocarcinoma
PCPG: Pheochromocytoma and Paraganglioma
PDK: phosphoinositide‐dependent kinase 1
PFI: progression‐free interval
PI3K: phosphoinositide‐3‐kinase
PIK3R1: phosphoinositide‐3‐kinase regulatory subunit 1
PIK3R2: phosphoinositide‐3‐kinase regulatory subunit 2
PIK3R3: phosphoinositide‐3‐kinase regulatory subunit 3
PIK3R5: phosphoinositide‐3‐kinase regulatory subunit 5
PIK3R6: phosphoinositide‐3‐kinase regulatory subunit 6
PIPs: phosphoinositides
PRAD: prostate adenocarcinoma
PTEN: phosphatase and TENsin homolog
qRT‐PCR: quantitative real‐time PCR
READ: rectum adenocarcinoma
RPPA: Reverse Phase Protein Array
RSE: read summarize experiment
RTK: receptor tyrosine kinase
SARC: sarcoma
Ser: serine
SH: Src homology
siRNA: small interfering ribonucleic acid
SKCM: Skin Cutaneous Melanoma
SNV: single nucleotide variant
STAD: stomach adenocarcinoma
TCGA: The Cancer Genome Atlas
TCGA‐CDR: TCGA Pan‐Cancer Clinical Data Resource
TGCT: Testicular Germ Cell Tumors
THCA: thyroid carcinoma
Thr: threonine
THYM: thymoma
UCEC: Uterine Corpus Endometrial Carcinoma
UCS: Uterine Carcinosarcoma
UCSC: University of California, Santa Cruz
UTR: untranslated region
UVM: Uveal Melanoma

## 1 Introduction

Phosphatidylinositol 3‐Kinases (PI3Ks), a family of lipid kinases, play a vital role in integrating and converting signals from growth factors, cytokines, and various environmental stimuli to regulate various cellular processes, including cell growth, proliferation, and survival [1, 2]. Based on their structure and function, PI3Ks are categorized into three classes (I–III), with class I being the most described [3, 4].

Class I enzymes exhibit a heterodimeric structure, comprising a catalytic subunit (p110) associated with a regulatory subunit (p85), and are subdivided into two subclasses: IA and IB. The class IA includes four highly homologous catalytic isoforms (p110α, p110β, and p110δ) which associate with any of the five p85‐regulatory isoforms, including p85α (along with its splicing variants p55α and p50α, encoded by *PIK3R1*), p85β (encoded by *PIK3R2*), or p55γ (encoded by *PIK3R3*) [5, 6]. In contrast, class IB PI3Ks consist of heterodimers composed of a p110γ catalytic subunit (encoded by *PIK3CG*) coupled with regulatory isoforms p101 (encoded by *PIK3R5*) or p87 (encoded by *PIK3R6*) [7].

Receptor tyrosine kinase (RTK) activation promotes PI3K localization to the plasma membrane, where p85α associates with p110α to promote the conversion of PIP_2_ to PIP_3_ [8, 9, 10, 11, 12]; PIP_3_ recruits Akt and its activating kinase PDK1 [13]. Akt is activated via phosphorylation by mTORC2 and PDK1 at Ser473 and Thr308, respectively, triggering protein synthesis through TSC, Rheb, and mTOR interactions [13, 14, 15, 16, 17, 18, 19], thereby enhancing various signaling pathways involved in cell survival, proliferation, growth, and cell cycle [20]. Conversely, the phosphatase PTEN deactivates the PI3K signaling by removing the phosphate group from the D‐3 position of phosphatidylinositol [21, 22, 23].

Aberrant PI3K signaling is a characteristic feature of different human cancers, highlighting the importance of the regulatory subunit *PIK3R1* in tumorigenesis. Alterations in *PIK3R1* expression, as well as mutations or deletions in *PIK3R1*, trigger the PI3K/AKT/mTOR pathway, further increasing cell growth, survival, proliferation, and migration [24, 25]. In normal tissues, *PIK3R1*/p85α is the predominant isoform; however, its expression is frequently reduced in cancer tissues [26, 27], indicating a tumor‐suppressive role of *PIK3R1*. Based on data from the cBioPortal for Cancer Genomics [28], *PIK3R1* ranks as the 11th most frequently mutated gene across 4429 tumors spanning 20 tumor types. Reduced expression of *PIK3R1*, mainly due to mutations or deletions, triggers the PI3K pathway and stimulates downstream AKT signaling, potentially inducing carcinogenesis [25, 29, 30, 31]. Thus, understanding the role of *PIK3R1* expression in cancer will help to shed light on the underlying molecular mechanisms underpinning *PIK3R*1‐induced tumorigenesis and pave the way for the development of therapeutic strategies.

We hypothesize that during the alternative splicing event (ASE), the *PI3KR1* is expressed as the p55α isoform, and such p55α modulates the PI3K pathway and triggers signaling events promoting malignancy through cell proliferation, growth, survival, motility, and metabolism. In the present study, the transcriptional expression levels of the regulatory isoform of *PIK3R1* p85α (long isoform) and its splicing variant, p55α (short isoform), in pan‐cancer were analyzed using the TCGA database. Additionally, we analyzed the correlation of gene expression with overall survival. In addition to the pan‐cancer analysis, we explored potential racial discrepancies (African American (AA) populations, compared to European American (EA) patients) in the expression of these isoforms. Although eleven Native American patients were included in the dataset, the sample size was too limited to allow for meaningful analysis. We also analyzed genetic alterations and expression levels of both p85α and p55α with the target players of the PI3K/Akt pathway.

## 2 Methods

### 2.1 Sample information

The original data of the public databases used for systematic pan‐cancer analysis of the regulatory isoform of *PIK3R1* p85α and its splicing variant, p55α, were mainly from The Cancer Genome Atlas (TCGA) (https://cancergenome.nih.gov/) (RRID:SCR_003193) for the 32 types of human cancers [32].

Below, provided are full names of the tumors alongside their corresponding abbreviations: adrenocortical carcinoma (ACC); bladder urothelial carcinoma (BLCA); breast invasive carcinoma (BRCA); cervical squamous cell carcinoma and endocervical adenocarcinoma (CESC); cholangiocarcinoma (CHOL); colon adenocarcinoma (COAD); lymphoid neoplasm diffuse large B‐cell lymphoma (DLBC); esophageal carcinoma (ESCA); glioblastoma multiforme (GBM); head and neck squamous cell carcinoma (HNSC); kidney chromophobe (KICH); kidney renal clear cell carcinoma (KIRC); kidney renal papillary cell carcinoma (KIRP); acute myeloid leukemia (LAML); brain lower grade glioma (LGG); liver hepatocellular carcinoma (LIHC); lung adenocarcinoma (LUAD); lung squamous cell carcinoma (LUSC); mesothelioma (MESO); ovarian serous cystadenocarcinoma (OV); pancreatic adenocarcinoma (PAAD); pheochromocytoma and paraganglioma (PCPG); prostate adenocarcinoma (PRAD); rectum adenocarcinoma (READ); sarcoma (SARC); skin cutaneous melanoma (SKCM); stomach adenocarcinoma (STAD); testicular germ cell tumors (TGCT); thyroid carcinoma (THCA); thymoma (THYM); uterine corpus endometrial carcinoma (UCEC); uterine carcinosarcoma (UCS); uveal melanoma (UVM).

### 2.2 Transcriptional expression analysis of the primary isoform of PIK3R1 (p85α) and its splicing variant (p55α)

We accessed RNA‐seq data from The Cancer Genome Atlas (TCGA) using the ‘recount3’ R package [33]. For each cancer type, junction read summarize experiment (RSE) objects were created and saved, and metadata were filtered to identify the primary tumor and its normal samples. The unique junction counts for the primary isoform (chr5:68281007–68292258:+) and the splicing variant isoform (chr5:68290835–68292258:+) of the *PIK3R1* gene were retrieved. These coordinates correspond to unique intronic regions specific to each isoform as annotated in the UCSC Genome Browser (https://genome.ucsc.edu) (RRID:SCR_005780) at the hg38 [34]. Expression levels were calculated by summing reads mapping to the borders of intronic regions, reflecting the expression of their corresponding isoforms (https://github.com/GaykalovaLab/PIK3R1_splicing).

Wilcoxon test was performed to compare expression levels between tumor and normal samples, and results were visualized using boxplots. Processed expression data for each cancer type was saved as CSV files. These files contained log‐transformed expression levels for both the primary isoform (p85α) and splicing variant (p55α) of *PIK3R1*, categorized by sample type (tumor or normal) and included sample barcodes for reference.

### 2.3 Transcriptional expression analysis of the primary isoform of PIK3R1 (p85α) and its splicing variant (p55α) based on racial disparity

Racial ethnicity across all 32 TCGA cancer types was obtained from the TCGA Pan‐Cancer Clinical Data Resource (TCGA‐CDR) [35]. Patients were classified into racial groups based on self‐reported ethnicity as recorded in the TCGA clinical data. For this study, we included the racial categories African American (AA) and European American (EA).

Based on the mean transcriptomic expression of the regulatory isoform of *PIK3R1* p85α, the patients were classified into ‘low’ and ‘high’ p85α expression. On the other hand, for the spliced variant, p55α, the patients were classified into ‘low’ expression if p55α expression = ‘0’ and were classified into ‘high’ expression if p55α expression > ‘0’. Based on the transcriptomic expression of p85α and p55α, the expression of isoforms was divided based on racial ethnicity and plotted against normal tissue.

### 2.4 Proteomic expression analysis

Protein expression analysis from Reverse Phase Protein Array (RPPA) data for the Pan‐Cancer cohort was obtained from the Genomic Data Commons Data Portal (https://gdc.cancer.gov/about‐data/publications/panimmune) (RRID:SCR_014514) [36] as well as the cBioPortal database (https://www.cbioportal.org/) (RRID:SCR_014555) [28]. Protein data for the target players of the PI3K/Akt pathway (AKT1/2/3, AKT, AKST_pS473, AKT_pT308, mTOR, mTOR_pS2448, p53, and PTEN) were analyzed. Protein expression for PIK3R1 was not available for PRAD and THCA, while protein expression data for PIK3R2 were not available for the KICH, KIRP, LIHC, PRAD, THCA, and UCEC cohorts in TCGA and were therefore not included in the analysis. For TP53, although it is an important tumor suppressor frequently altered in cancer, RPPA‐based protein expression data for TP53 were not available for the LIHC cohort. For UCEC, while the cohort in our analysis did not correspond to the UCEC cohort for the RPPA data available for TP53 expression, protein expression for p110a was not available and, hence, was not plotted.

Based on the mean transcriptomic expression of the regulatory isoform of *PIK3R1* p85α, the patients were classified into ‘low’ and ‘high’ p85α expression. On the other hand, for the spliced variant, p55α, the patients were classified into ‘low’ expression if p55α expression = ‘0’ and were classified into ‘high’ expression if p55α expression > ‘0’. Based on the transcriptomic expression of p85α and p55α, the expression of the target players of the PI3K/Akt pathway was divided and plotted.

### 2.5 Survival prognosis analysis

Kaplan–Meier curves were generated to estimate the probability of survival over time for the entire cohort. Survival data across all 32 TCGA cancer types were obtained from the TCGA Pan‐Cancer Clinical Data Resource (TCGA‐CDR) [35]. In addition, patients were classified into racial groups according to self‐reported ethnicity available in the TCGA clinical data and included classifications such as AA and EA.

Based on the mean transcriptomic expression of the regulatory isoform of *PIK3R1* p85α, the patients were classified into ‘low’ and ‘high’ p85α expression. On the other hand, for the spliced variant, p55α, the patients were classified into ‘low’ expression if p55α expression = ‘0’ and were classified into ‘high’ expression if p55α expression > ‘0’. Based on the expression of p85α and p55α, patients were classified into two groups, and the overall survival (OS) and progression‐free interval (PFI) were analyzed. Likewise, Kaplan–Meier survival curves were also generated to compare survival outcomes between the low and high p85α expression groups, stratified by racial categories.

Multivariate Cox proportional hazards regression analyses were performed with the survival, survminer, and forest plot R packages to evaluate the associations between OS and PFI with various clinical parameters, as well as the expression of *PIK3R1* p85α and its spliced variant, p55α.

### 2.6 Genetic alteration analysis

Genetic variants present in the primary and splicing variant of *PIK3R1* (p85α and p55α, respectively), including alteration frequency, type, and site of mutation, and any relevant clinical significance and association with disease, were obtained from the Genome Aggregation Database (gnomAD v4.1.0) (gnomad.broadinstitute.org) (RRID:SCR_014964) [37]. The dataset available in gnomAD encompasses a total of 730 947 exome sequences and 76 215 whole‐genome sequences from unrelated individuals’ sequences as part of diverse disease‐specific and population genetic investigations, totaling 807 162 subjects, and is aligned against the GrCh38.p14 reference genome. The dataset comprises a total of ~786.5 million single nucleotide variants (SNVs) and 122 million InDels from 730 947 exomes. Within gnomAD, structural variants are classified as genomic rearrangements that encompass a minimum of 50 base pairs of DNA. For consequent analyses, variants were classified according to gnomAD annotations, including 5′ untranslated region (UTR), 3′UTR, intronic, splice region, splice donor, missense, start lost, start gained, stop, frameshift, splice, missense, synonymous, and in‐frame insertion classifications.

For genetic variant analysis, we used the coordinates (5:68,290,834‐68,281,006 and 5:68,290,834‐68,292,259 GRCh38) for p85α and p55α, respectively.

### 2.7 Cell culture

The human LUSC cell line, NCI‐H358 (RRID:CVCL_1559), was provided by Dr. David Sidransky from Johns Hopkins University, Baltimore. The H358 (RRID:CVCL_1559) cells were cultured in RPMI‐1640 media (Invitrogen, Carlsbad, CA, USA) supplemented with 10% Fetal Bovine Serum (GeminiBio, West Sacramento, CA, USA) mixed with X1 Penicillin and Streptomycin (Corning, NY, USA). All cultured cell growth occurred in a 5% CO_2_ incubator at 37 °C. Cell lines enumerated in Table S1 were analyzed for p85α and p55α expression using available RNA from our previous publication [38]. The NCI‐H series cell lines were originally established by the National Cancer Institute. Each cell line was authenticated using a Short Tandem Repeat Identifier kit (Applied Biosystems, Foster City, CA, USA) and tested negative for mycoplasma contamination.

### 2.8 Quantitative real‐time PCR (qRT‐PCR)

RNA isolation was performed using Qiazol and RNeasy Kit (Qiagen, Redwood City, CA, USA) as per the manufacturer's protocol. Reverse transcription was performed with the MultiScribe Reverse Transcription kit (Invitrogen). RNA expression was determined using TaqMan qRT‐PCR using 0.6% Platinum Taq DNA Polymerase (Invitrogen), 2% ROX Reference Dye (Invitrogen), 0.2 mm of dNTPs (our laboratory), 0.6 μm of each primer, and 0.33 μm of probe per reaction on 25 ng·μL^−1^ of DNA template with the following primer‐probe sets: Forward Primer CAGCAGCCAGCTCTGATAAT, Reverse Primer TCATACCGTTGTTGGCTACAG, and probe GAGGCAGTGCTGGTGCAGG were used for the detection of the primary isoform of *PIK3R1*, p85α. Forward Primer GGAATATGGAAGACCTGGATTTAGA, Reverse Primer TCATACCGTTGTTGGCTACAG, and probe GAGGCAGTGCTGGTGGGTC were used for the detection of the splicing variant of *PIK3R1*, p55α. All assays were quantified in triplicate against a *GAPDH* control 20X Gene Expression Assay (Hs02758991_g1) (Invitrogen) using the 2^−ΔΔCT^ method [39].

### 2.9 siRNA transfection of cell lines

The custom siRNAs for the primary isoform (p85α) (Sense: 5′‐AGGGAAGAAGUGAAUGAAAUU‐3′ and Antisense: 5′‐UUUCAUUCACUUCUUCCCUUU‐3′) and for the splicing variant (p55α) (Sense: 5′‐GUACAAUACUGUUUGGAAUUU‐3′ and Antisense: 5′‐AUUCCAAACAGUAUUGUACUU‐3′) were obtained from Dharmacon (Lafayette, CO, USA). Transfection of single siRNAs was performed in Opti‐MEM (Invitrogen) using RNAiMAX Lipofectamine Reagent (Invitrogen) in parallel with ON‐TARGETplus Pool (042412) controls.

The cells were plated and cultured in 6‐well plates and transfected in reduced‐serum media (Opti‐MEM, Gibco, Waltham, MA, USA) with the 20 μm siRNA, using Lipofectamine RNAi‐MAX reagent (Invitrogen) for 16 h. The transfected medium was replaced with complete medium with necessary supplements (see above) after 16 h of transfection as per the manufacturer's protocol. Cells were harvested after 72‐h post‐transient knockdown for RNA isolation. The transfection efficiency and the level of the endogenous gene expression were monitored by qRT‐PCR.

### 2.10 Cell proliferation assay

Alamar Blue assay (Bio‐Rad, Hercules, CA, USA) was used to determine cell viability according to the manufacturer's protocol. The cell line, H358, was treated with Alamar Blue (Bio‐Rad) diluted 1:10 in Opti‐MEM Media (Gibco) as described previously [40]. Fluorescence intensity was measured at 0 h (before transfection) to establish the baseline viability at an excitation/emission wavelength of 530/590 nm on a Spectramax M5 microplate reader. Cells were then transfected with the respective siRNA as described above. Post‐transfection, Alamar Blue reagent was added to each well, and fluorescence intensity was measured at 24, 48, and 72 h after transfection.

### 2.11 BaseScope assay

BaseScope™ Assay is used to detect short RNA target sequences (50–300 nucleotides) and can detect splice variants, exon junctions, and point mutations in addition to pre‐miRNA and circular RNA [41].

We utilized BaseScope™ to detect the isoforms of *PIK3R1* (p85α and p55α) as per the manufacturer's guidelines (BaseScope™ Detection Reagent Kit‐RED, Cat# 322900) (Advanced Cell Diagnostics (ACD), Newark, CA, USA). A 1zz BaseScope™ Duplex probe targeting the isoforms of *PIK3R1* was designed (ACD, Newark, CA, USA). For the controls, a Hs‐1zz BaseScope™ Duplex Control Probe (ACD, Cat# 700101) and a 3zz BaseScope™ Duplex Negative Control Probe (ACD, Cat# 700151) were used. Chromogenic detection was done using the BaseScope Fast RED, followed by counterstaining with Hematoxylin solution Gill I (VWR International, Radnor, PA, USA).

The signals were observed, and images were captured using the Lumenera INFINITY3 camera (Lumenera, Ottawa, ON, CA) at 20× magnification (Nikon Eclipse E600 Microscope, Nikon, Melville, NY, USA) and analyzed using the CellProfiler Cell Image Analysis Software (www.cellprofiler.org) [42]. Color deconvolution and feature enhancement techniques were applied to identify cell nuclei, red punctates, and green punctates. Nuclei were expanded by 50 pixels to define cell boundaries. These expanded boundaries were then used to mask and isolate the red and green punctates within each cell. The number of red and green punctates inside the masked cell borders was quantified. Finally, histograms were generated to depict the distribution of cells based on the number of punctates they contained.

### 2.12 Statistical analysis

Graphs were plotted using GraphPad Prism Software (version 10.00; GraphPad Software, San Diego, CA, USA) (RRID:SCR_002798). For the transcriptional expression analysis of the primary isoform of *PIK3R1* and its splicing variant, the Wilcoxon test was performed to compare expression levels between tumor and normal samples, and results were visualized using boxplots. A one‐way ANOVA statistical analysis was used to compare the transcriptional expression analysis based on racial ethnicity against normal samples. Log‐rank tests were employed to compare survival curves between subgroups based on the ‘low’ and ‘high’ expressions of p85α and p55α. Two‐way ANOVA, followed by Sidak's multiple comparison tests, was used to compare the differences between the scrambled siRNA (control), p85α siRNA and p55α siRNA at different time points to compare cell proliferation rates. Correlation of protein expression was analyzed using two‐way ANOVA statistical analysis. Statistical value *P* < 0.05 was considered significant.

## 3 Results

### 3.1 Transcriptional expression analysis of the primary isoform of PIK3R1 (p85α) and its splicing variant (p55α)

To investigate isoform‐specific expression changes of *PIK3R1* in cancer, transcriptomic analysis of the expression levels of the primary isoform p85α and the alternatively spliced variant p55α across 32 TCGA cancer types by comparing tumor and matched normal tissues (Fig. S1). Data revealed loss of p85α expression in the tumor samples as compared to the normal tissue samples (Fig. S1). On the other hand, p55α expression was upregulated in the tumor samples as compared to the normal tissue samples (Fig. S1). Data for normal tissue were not available for ACC, DLBC, LGG, MESO, OV, TGCT, THYM, UCS, and UVM and were excluded from further analysis.

**Fig. 1:**
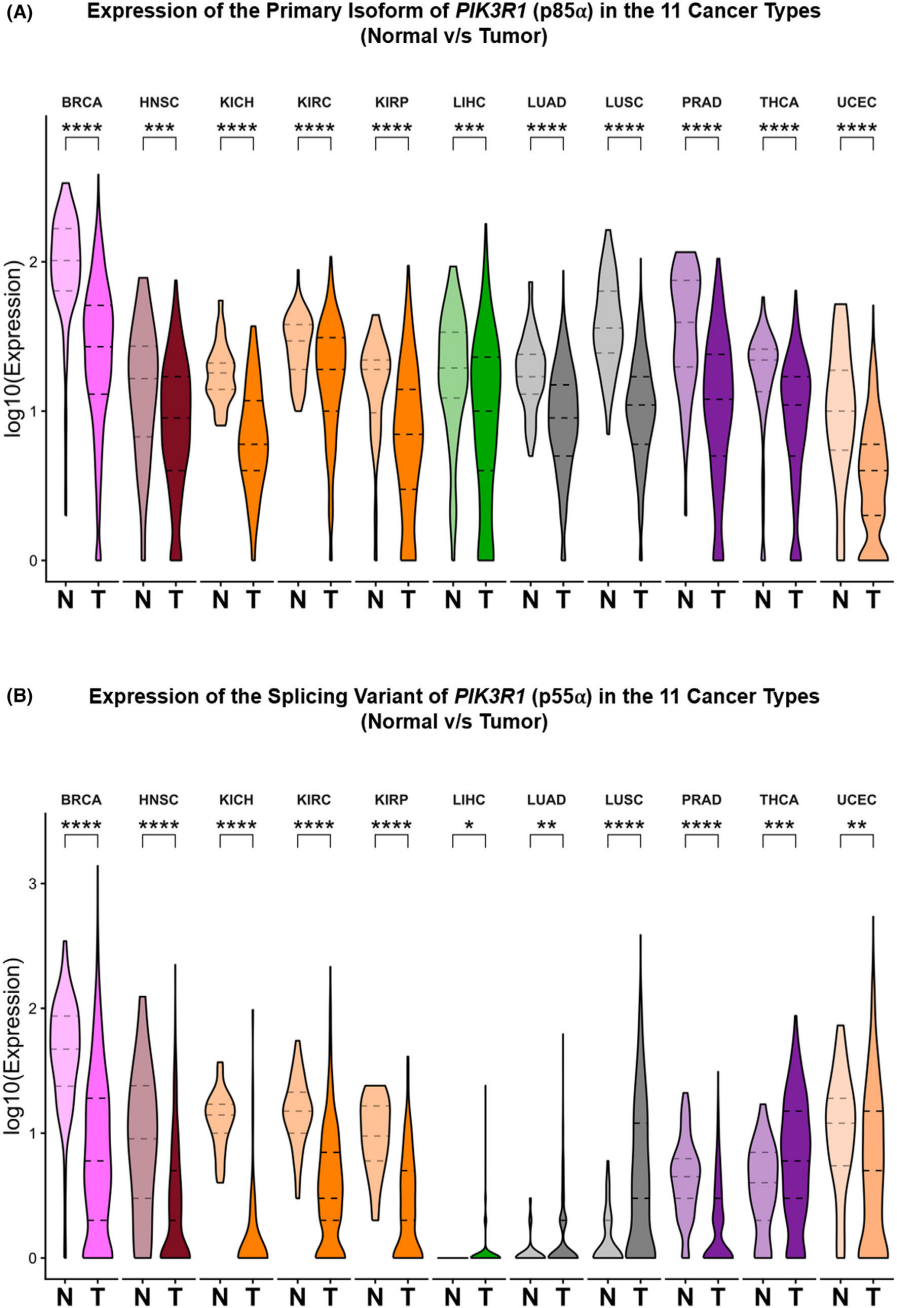
Transcriptional expression analysis of the (A) primary isoform of *PIK3R1* (p85α) and (B) its splicing variant (p55α). The violin plot illustrates the transcriptional expression analysis of the primary isoform of *PIK3R1*, p85α, and its splicing variant, p55α, in the 11 cancer types analyzed between the normal and tumor tissue samples. The x‐axis denotes the tissue type, while the y‐axis indicates the relative expression level of the isoform. See also Fig. S1. Statistical significance is denoted by asterisks: *P < 0.05, **P < 0.01, ***P < 0.001, ****P < 0.0001. N, Normal tissue; T, Tumor tissue; BRCA, Breast invasive carcinoma (BRCA); HNSC, Head and Neck squamous cell carcinoma; KICH, Kidney Chromophobe; KIRC, Kidney renal clear cell carcinoma; KIRP, Kidney renal papillary cell carcinoma; LIHC, Liver hepatocellular carcinoma; LUAD, Lung adenocarcinoma; LUSC, Lung squamous cell carcinoma; PRAD, Prostate adenocarcinoma; THCA, Thyroid carcinoma; UCEC, Uterine Corpus Endometrial Carcinoma.

Of the 32 cancers, only 11 of them (BRCA, HNSC, KICH, KIRC, KIRP, LIHC, LUAD, LUSC, PRAD, THCA, and UCEC) had a significant expression of p85α and p55α in comparison to the normal tissue samples (Fig. 1). Notably, p85α expression was significantly downregulated in tumor tissues compared to normal tissues across the 11 TCGA cancer types (BRCA, HNSC, KICH, KIRC, KIRP, LIHC, LUAD, LUSC, PRAD, THCA, and UCEC), suggesting a potential loss of its canonical regulatory function in tumorigenesis (Fig. 1A). In contrast, p55α exhibited a distinct expression pattern, with significant upregulation in tumor tissues observed in several cancers, including LIHC, LUSC, THCA, and UCEC (Fig. 1B). This isoform‐specific shift, characterized by suppression of p85α and concomitant increase in p55α, suggests a potential isoform switching mechanism in tumors, possibly favoring the expression of the shorter p55α with distinct regulatory roles. These findings highlight dysregulation of *PIK3R1* isoform expression in cancer, which can plausibly contribute to altered PI3K signaling and tumor progression.

**Fig. 2:**
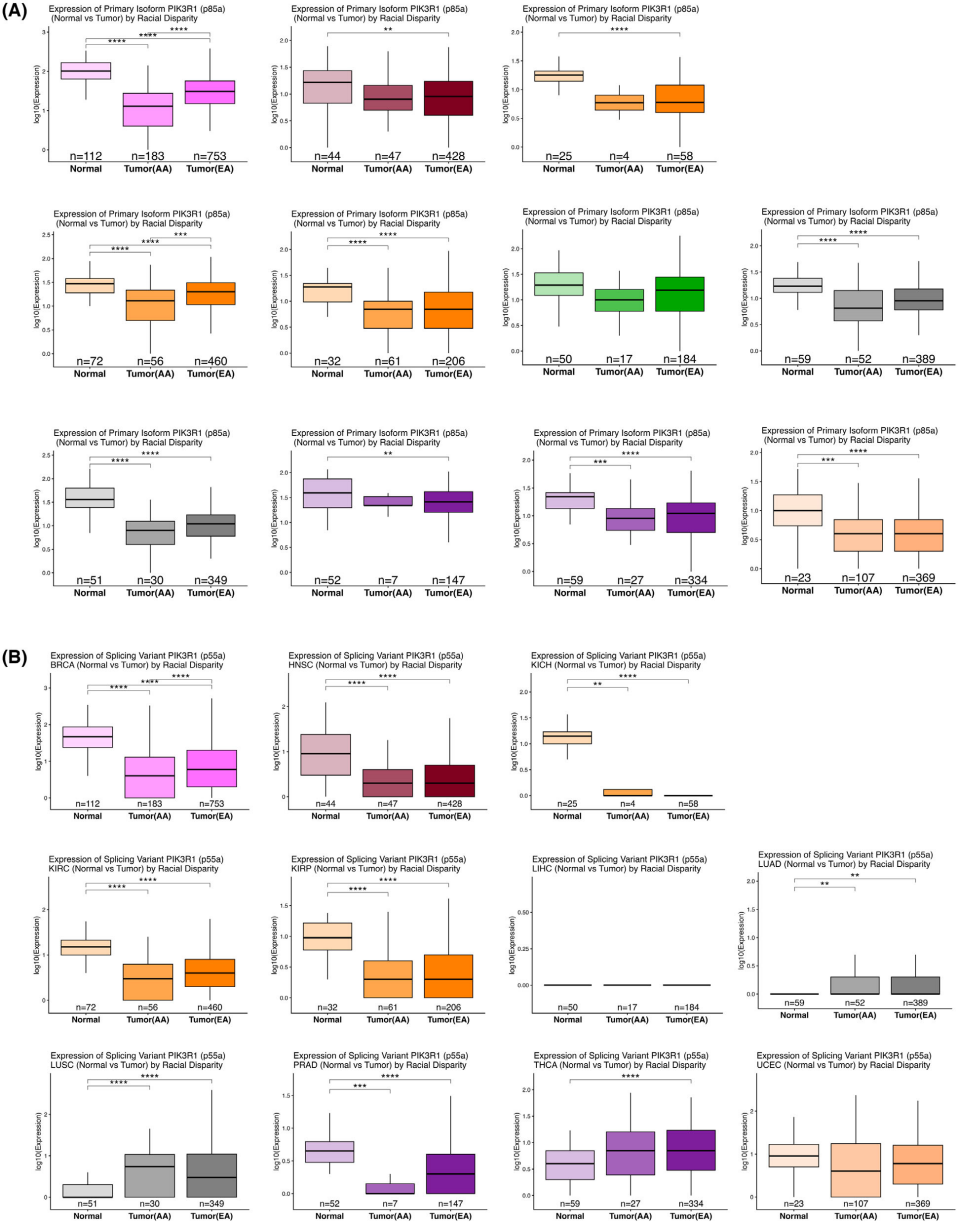
Differential expression of the (A) Primary Isoform of *PIK3R1* (p85α) and (B) splicing variant of *PIK3R1* (p55α) in the 11 TCGA cancer types based on racial disparity. Box plots represent the expression levels (log_10_ transformed) of the primary isoform of *PIK3R1* (p85α) and its splicing variant (p55α) between normal tissue and tumor samples from African American (AA) and European American (EA) cohorts across the significant 11 TCGA cancer types (BRCA, HNSC, KICH, KIRC, KIRP, LIHC, LUAD, LUSC, PRAD, THCA, and UCEC), showing the median (center line), interquartile range (box), and 1.5 × IQR whiskers to illustrate expression distributions. (A) In BRCA and KIRC, there is a significant downregulation of p85α in tumors from both AA and EA patients compared to normal tissue, with EA patients showing a more pronounced reduction. Differences between AA and EA tumor expression are also significant. In HNSC, KICH, KIRP, LUAD, LUSC, THCA, and UCEC, tumor samples from both AA and EA patients show significant downregulation of p85α compared to normal tissues, with EA tumors exhibiting a slightly more pronounced reduction. In PRAD, while p85α expression is significantly reduced in EA tumor samples compared to normal tissues, the expression in AA tumors is not significantly different from normal tissues, highlighting a potential racial discrepancy in prostate cancer. However, in LIHC, the expression of p85α is not significantly different between tumor and normal tissues for either AA or EA patients, indicating limited racial disparity regarding p85α expression. (B) In BRCA, there is a significant downregulation of p55α in tumors from both AA and EA patients compared to normal tissue, with EA patients showing a more pronounced reduction. In KICH, LUAD, KIRC and LUSC and THCA, p55α expression is significantly elevated in tumors from AA patients compared to normal tissue, while no significant changes are seen in EA patients, suggesting a racial disparity in these cancers. On the contrary, no significant differences are observed in p55α expression between normal and tumor tissues for either AA or EA patients in LIHC and UCEC, suggesting limited racial disparity. This figure highlights the impact of racial disparity on the differential expression of *PIK3R1* isoforms in tumors relative to normal tissues, revealing significant variations in expression patterns between AA and EA patients in multiple cancer types. Statistical significance was determined using one‐way ANOVA statistical tests, with significance levels indicated by asterisks (*P < 0.05, **P < 0.01, ***P < 0.001, ****P < 0.0001). AA, African American; EA, European American. BRCA, Breast invasive carcinoma (BRCA); HNSC, Head and Neck squamous cell carcinoma; KICH, Kidney Chromophobe; KIRC, Kidney renal clear cell carcinoma; KIRP, Kidney renal papillary cell carcinoma; LIHC, Liver hepatocellular carcinoma; LUAD, Lung adenocarcinoma; LUSC, Lung squamous cell carcinoma; PRAD, Prostate adenocarcinoma; THCA, Thyroid carcinoma; UCEC, Uterine Corpus Endometrial Carcinoma.

In addition to the pan‐cancer analysis, we explored potential racial discrepancies in the expression of these isoforms in the 11 significant TCGA cancer types (Fig. 2). The primary isoform of *PIK3R1* (p85α) was significantly different between normal and EA samples in BRCA, HNSC, KICH, KIRC, KIRP, LUAD, LUSC, PRAD, THCA, and UCEC (Fig. 2A). On the other hand, while the splicing variant of *PIK3R1* (p55α) was significantly different between normal and EA samples in BRCA, HNSC, KICH, KIRC, KIRP, LUAD, LUSC, PRAD and THCA (Fig. 2B), significance between normal and AA samples was observed in BRCA, HNSC, KICH, KIRC, KIRP, LUAD, LUSC, and PRAD (Fig. 2B). However, while only p85α showed a significant difference between AA and EA racial/ethnic groups in BRCA and KIRC, p55α expression was significantly different between the AA and EA racial/ethnic groups only in BRCA (Fig. 2).

### 3.2 Correlation between the expression levels of the primary isoform of PIK3R1 (p85α) and its splicing variant (p55α) with patient outcome

**Fig. 3:**
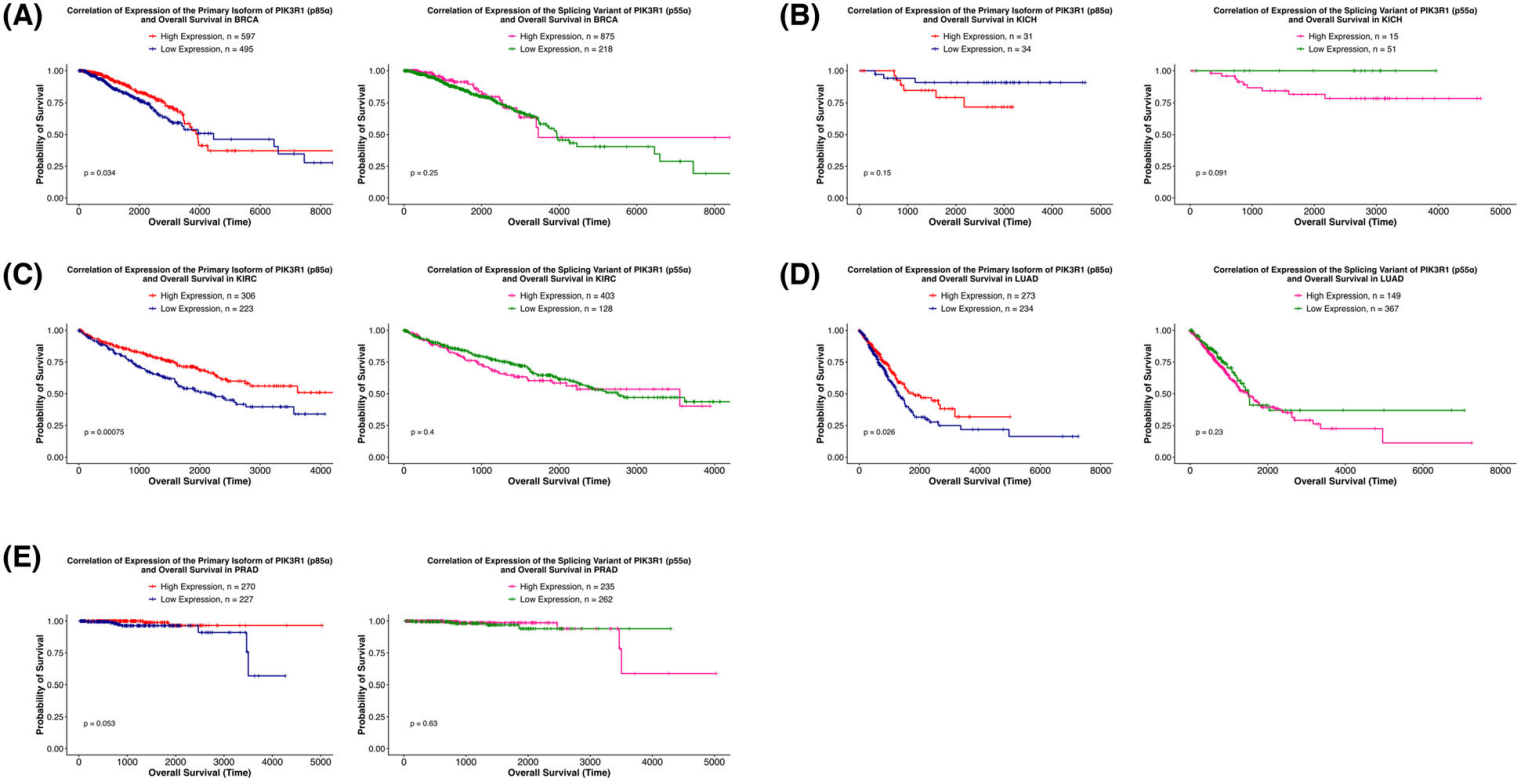
Correlation between the expression levels of the primary isoform of *PIK3R1* (p85α) and splicing variant of *PIK3R1* (p55α) with overall survival (OS). The figure displays Kaplan–Meier survival curves illustrating the correlation between expression levels of the primary isoform of *PIK3R1*, p85α, and its splicing variant, p55α, with overall survival across (A) BRCA, (B) KICH, (C) KIRC, (D) LUAD, and (E) PRAD. Each curve represents how the survival probability changes over time, with the x‐axis denoting time in days and the y‐axis representing the proportion of patients surviving. Curves are stratified based on the mean expression of the primary isoform of *PIK3R1*, p85α. On the other hand, for the spliced variant, p55α, curves are stratified based on p55α expression = ‘0’ or > ‘0’ Statistical significance is determined using log‐rank tests. The divergence or convergence of the curves indicates the potential impact of p85α or p55α expression on overall survival outcomes in BRCA, KICH, KIRC, LUAD, and PRAD. Low p85α expression significantly correlated with poor OS in BRCA, KICH, KIRC, LUAD, and PRAD, while the correlation between high p55α expression and OS was found only in KICH. See also Fig. S3. BRCA, Breast invasive carcinoma; KICH, Kidney Chromophobe; KIRC, Kidney renal clear cell carcinoma; LUAD, Lung adenocarcinoma; PRAD, Prostate adenocarcinoma.

We then performed survival analysis to correlate the expression of both the *PIK3R1* isoforms, p85α and p55α, and OS for the 11 significant TCGA cancer types (Fig. S2). Low p85α expression significantly correlated with poor OS in BRCA, KIRC, LUAD, and PRAD (Fig. 3A,C,D,E), while although a correlation between p55α expression and OS was found only in KICH (Fig. 3B), it had not reached significance. This inverse relationship suggests that these isoforms may have functionally distinct or even opposing roles in tumor biology and patient prognosis.

**Fig. 4:**
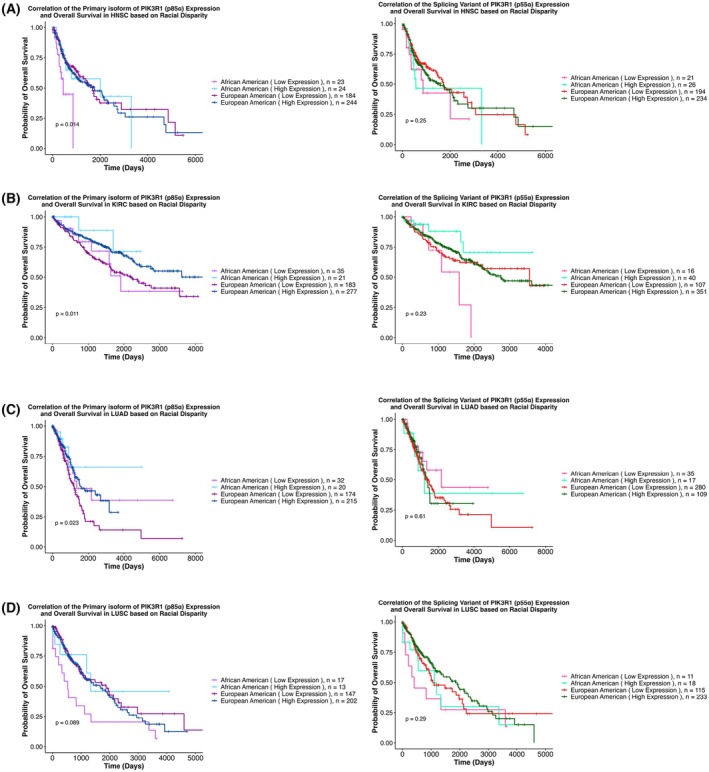
Correlation between the expression levels of the primary isoform of *PIK3R1* (p85α) and splicing variant of *PIK3R1* (p55α) with overall survival (OS) based on racial disparity. The figure displays Kaplan–Meier survival curves illustrating the correlation between expression levels of the primary isoform of *PIK3R1*, p85α, and its splicing variant, p55α, with overall survival across (A) HNSC, (B) KIRC, (C) LUAD, and (D) LUSC, categorized by racial groups. Each curve represents how the survival probability changes over time, with the x‐axis denoting time in days and the y‐axis representing the proportion of patients surviving. Curves are stratified based on the mean expression of the primary isoform of *PIK3R1*, p85α. On the other hand, for the spliced variant, p55α, curves are stratified based on p55α expression = ‘0’ or > ‘0’. The graph includes four distinct lines representing different patient groups, with each line depicting the probability of overall survival over time for the respective group. Statistical significance is determined using log‐rank tests. The divergence or convergence of the curves indicates the potential impact of p85α or p55α expression on overall survival outcomes in HNSC, KIRC, LUAD, and LUSC based on racial disparity. See also Fig. S3. HNSC, Head and Neck squamous cell carcinoma; KIRC, Kidney renal clear cell carcinoma; LUAD, Lung adenocarcinoma; LUSC, Lung squamous cell carcinoma.

The differential expression of the isoforms (p85α and p55α) based on race was further analyzed with disparities in OS (Fig. S3). In HNSC, KIRC, and LUAD, data indicated that low expression of p85α correlated with poor OS in both racial groups (Fig. 4A,B,C). For p85α expression, similar trends were observed in LUSC, falling just below the threshold of statistical significance (*P* = 0.08, Fig. 4D). Significant survival disparities when stratified by p55α expression were not observed in HNSC, KIRC, LUAD, and LUSC (Fig. 4A–D).

**Fig. 5:**
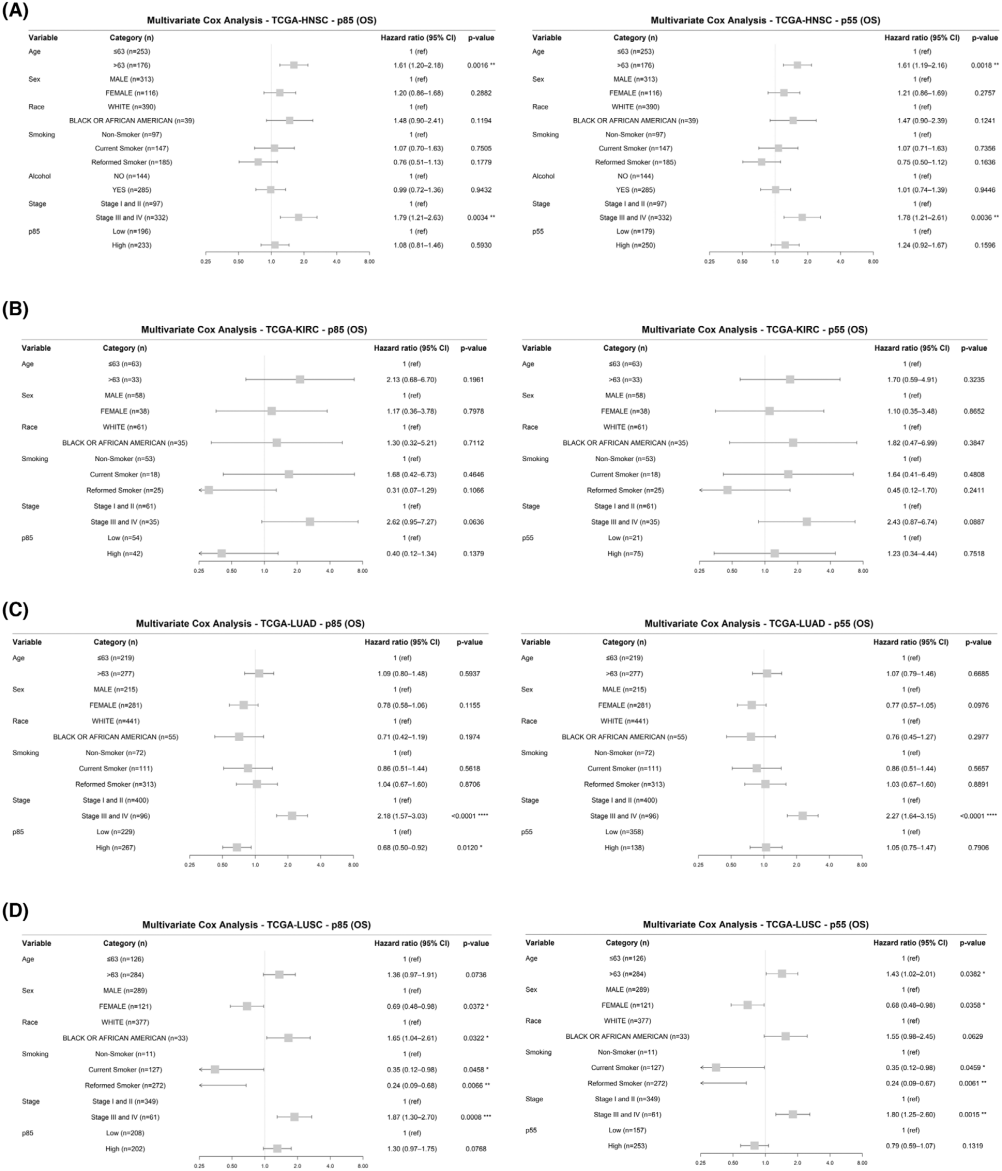
Multivariate cox analysis identifying factors affecting overall survival. The figure displays forest plots for hazard ratios from the Cox proportional hazard multivariate models, illustrating the correlation between expression levels of the primary isoform of *PIK3R1*, p85α, and its splicing variant, p55α, with progression‐free intervals across (A) HNSC, (B) KIRC, (C) LUAD, and (D) LUSC adjusting for age, sex, race, stage, smoking history, and alcohol history. The squares indicate hazard ratios and horizontal bars represent 95% confidence intervals; the vertical line at HR = 1 denotes no effect on survival. Statistical significance is denoted by asterisks: *P < 0.05, **P < 0.01, ***P < 0.001, ****P < 0.0001. HNSC, Head and Neck squamous cell carcinoma; KIRC, Kidney renal clear cell carcinoma; LUAD, Lung adenocarcinoma; LUSC, Lung squamous cell carcinoma.

To further examine if overall survival varies by race and p85α or p55α expression, we performed a multivariate Cox analysis to adjust for race as well as age, sex, stage, smoking history, alcohol consumption, and either p85α or p55α expression. Multivariate Cox analysis revealed no significant association between race and overall survival in HNSC, KIRC, and LUAD, but high expression of p85α in KIRC and LUAD is associated with a decrease in relative risk of mortality (Fig. 5A–C). Additionally, in HNSC and LUAD, advanced stage is associated with increased risk of mortality, regardless of p85α or p55α expression (Fig. 5A,C). Moreover, in HNSC, older patients have an increased risk of mortality (Fig. 5A). In LUSC, AAs have a 65% increase in relative risk of mortality compared to EAs with similar p85α expression (HR = 1.65, CI = 1.04–2.61, *P* = 0.0322). Although just below the threshold of significance, a similar trend is observed for p55α expression, with AAs having a 55% increase in relative risk of mortality compared to EAs (HR = 1.55, CI = 0.98–2.45, *P* = 0.0629). Smoking status, stage, and sex are also associated with overall survival (Fig. 5D). Together, these results indicate that in LUSC, race plays a significant role in overall survival, and in KIRC and LUAD, p85α expression may be an independent prognostic factor.

**Fig. 6:**
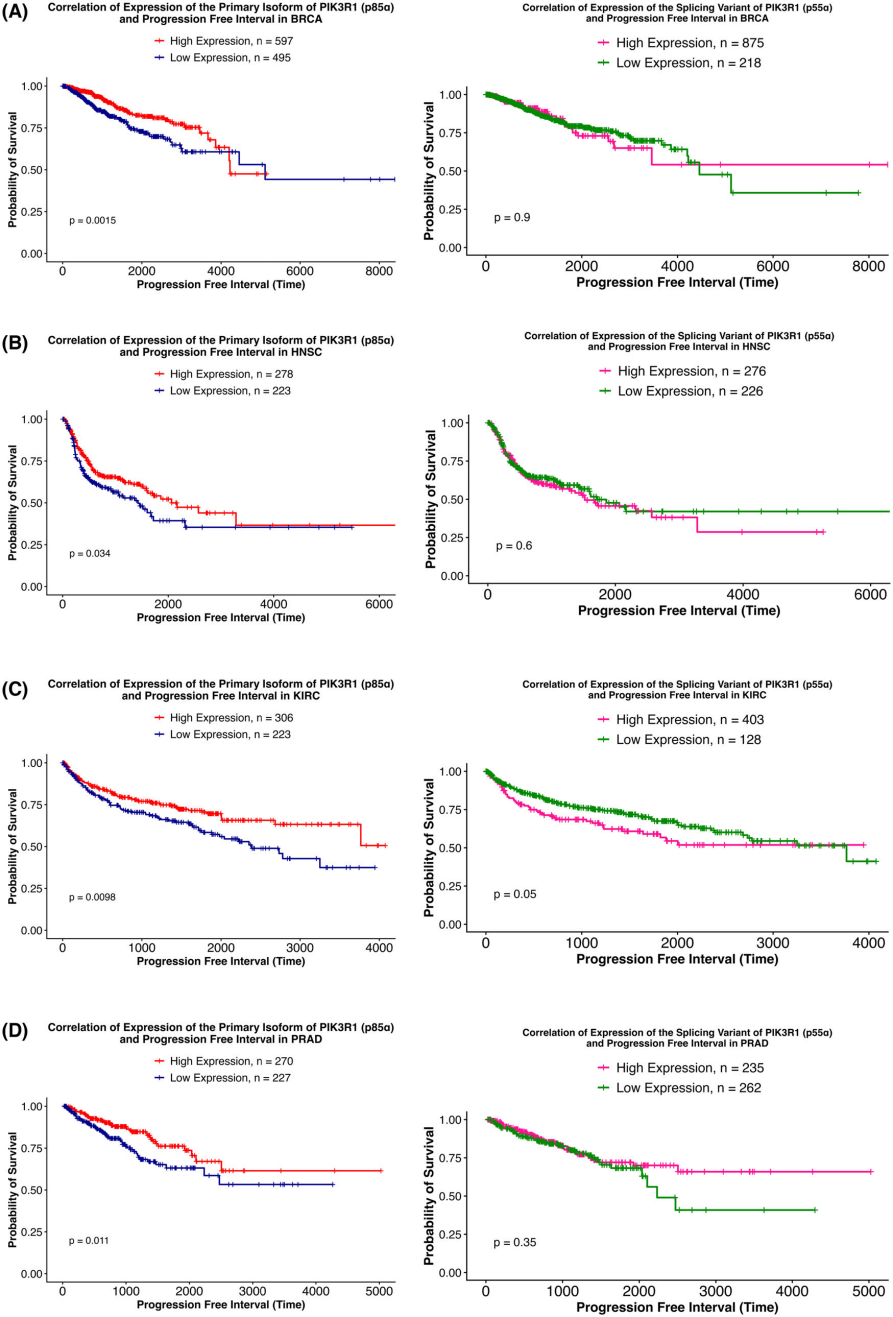
Correlation between the expression levels of the primary isoform of *PIK3R1* (p85α) and splicing variant of *PIK3R1* (p55α) with progression‐free interval (PFI). The figure displays Kaplan–Meier survival curves illustrating the correlation between expression levels of the primary isoform of *PIK3R1*, p85α, and its splicing variant, p55α, with progression‐free intervals across (A) BRCA, (B) HNSC, (C) KIRC, and (D) PRAD. Each curve represents how the probability of recurrence changes over time, with the x‐axis denoting time in days and the y‐axis representing the proportion of patients who have not experienced disease progression or recurrence. Curves are stratified based on the mean expression of the primary isoform of *PIK3R1*, p85α. On the other hand, for the spliced variant, p55α, curves are stratified based on p55α expression = ‘0’ or >‘0’. Statistical significance is determined using log‐rank tests. The divergence or convergence of the curves indicates the potential impact of p85α or p55α expression on PFI outcomes in different cancer types. Low p85α expression significantly correlated with poor PFI in BRCA, HNSC, and KIRC, while high p55α expression correlated with PFI in KIRC. See also Fig. S4. BRCA, Breast invasive carcinoma (BRCA); HNSC, Head and Neck squamous cell carcinoma; KIRC, Kidney renal clear cell carcinoma; PRAD, Prostate adenocarcinoma.

We further analyzed the correlation between the *PIK3R1* isoforms, p85α and p55α, and progression‐free interval (PFI) in the 11 TCGA cancer types (Fig. S4). Of the 11 cancer types, while low p85α expression correlated with PFI in BRCA, HNSC, KIRC, and PRAD (Fig. 6A–D), high p55α expression correlated with PFI in KIRC (Fig. 6C).

**Fig. 7:**
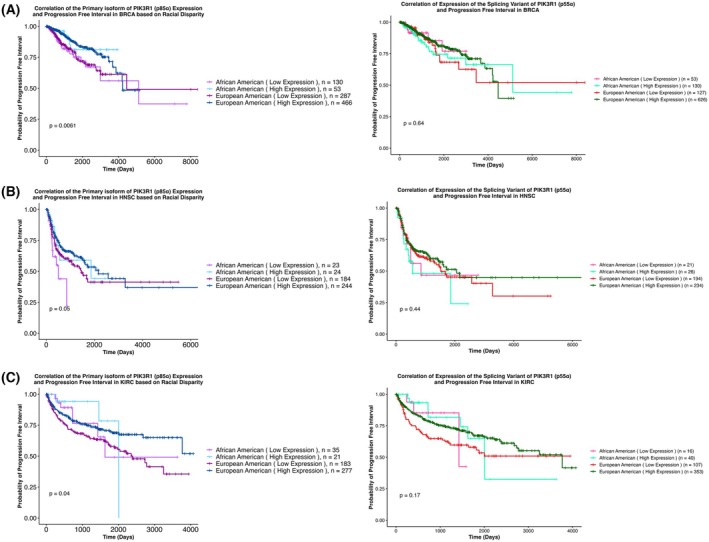
Correlation between the expression levels of the primary isoform of *PIK3R1* (p85α) and its splicing variant (p55α) with progression‐free interval (PFI) based on racial disparity. The figure displays Kaplan–Meier survival curves illustrating the correlation between expression levels of the primary isoform of *PIK3R1*, p85α, and its splicing variant, p55α, with progression‐free intervals across (A) BRCA, (B) HNSC, and (C) KIRC categorized by racial groups. Each curve represents how the probability of recurrence changes over time, with the x‐axis denoting time in days and the y‐axis representing the proportion of patients who have not experienced disease progression or recurrence. Curves are stratified based on the mean expression of the primary isoform of *PIK3R1*, p85α. On the other hand, for the spliced variant, p55α, curves are stratified based on p55α expression = ‘0’ or >‘0’. The graph includes four distinct lines representing different patient groups, with each line depicting the probability of overall survival over time for the respective group. Statistical significance is determined using log‐rank tests. The divergence or convergence of the curves indicates the potential impact of p85α or p55α expression on PFI outcomes in BRCA, HNSC, and KIRC based on racial disparity. See also Fig. S5. BRCA, Breast invasive carcinoma; HNSC, Head and Neck squamous cell carcinoma; KIRC, Kidney renal clear cell carcinoma.

Moreover, the differential expression of the isoforms (p85α and p55α) based on race was further analyzed with disparities in PFI (Fig. S5). For PFI outcome, correlation of low p85α expression and poor outcome was found in BRCA, HNSC, and KIRC for both race groups (Fig. 7A–C). In LUSC, high expression of p55α in EAs correlated with poor PFI, although it did not reach significance (*P* = 0.069) (Fig. 7D).

Multivariate Cox analysis revealed no significant associations with race and PFI in BRCA, HNSC, and KIRC (Fig. S6). Moreover, in BRCA, high expression of p85α is associated with a 33% decrease in the relative risk of disease progression (HR = 0.67, CI = 0.48–0.92, *P* = 0.0150) (Fig. S6A). Analysis further showed that advanced stage is associated with increased disease progression in BRCA, HNSC, and KIRC (Fig. S6). In HNSC, alcohol consumption is associated with an increase in relative disease progression (Fig. S6B). Together, these data suggests other factors, such as advanced stage and substance use, may contribute to observed variations in cancer prognosis rather than race.

### 3.3 Functional validation of the primary isoform (p85α) and splicing variant (p55α) of PIK3R1

We proceeded to perform validation studies using lung cancer cell lines. Our aim was to functionally characterize the roles of the p85α and p55α in cancer models.

**Fig. 8:**
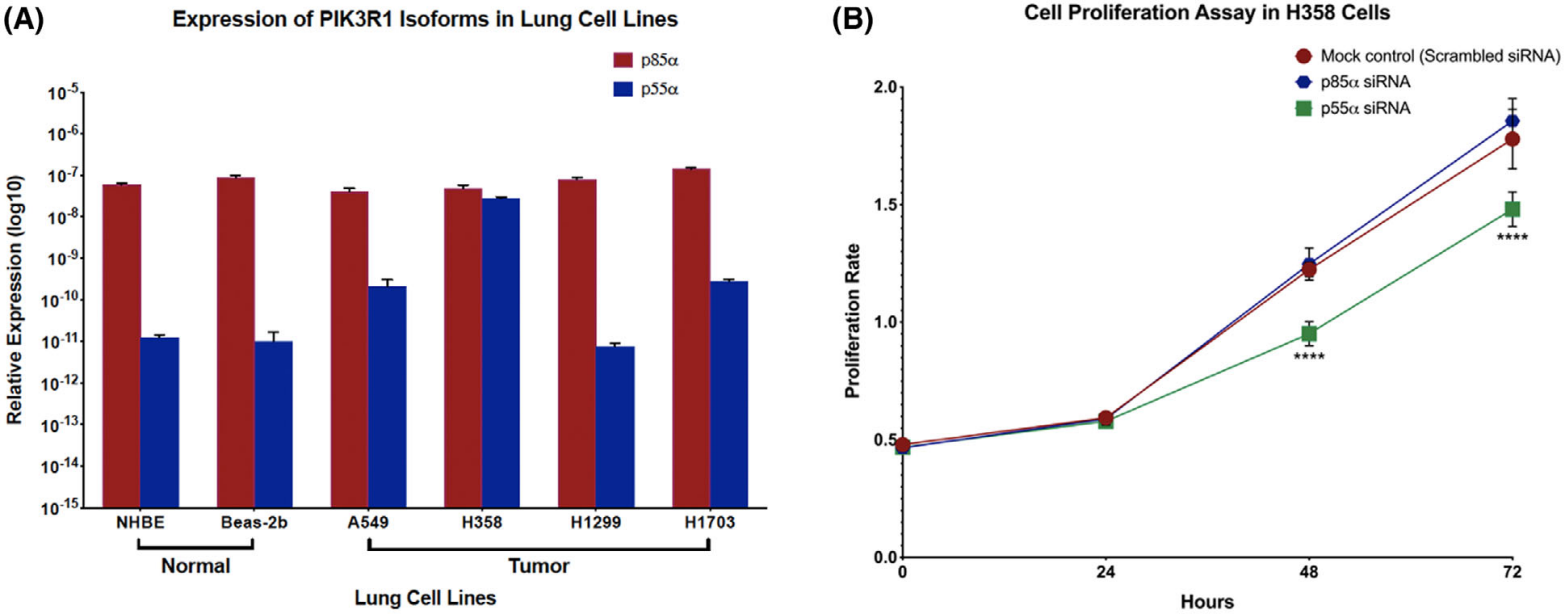
(A) Differential expression of the *PIK3R1* isoforms (p85α and p55α) in a panel of lung cell lines. qRT‐PCR was performed in lung cell lines to measure the relative mRNA expression levels of the two PIK3R1 isoforms; p85α (red) and p55α (blue). Expression was measured in 2 normal bronchial epithelial cell lines (NHBE, Beas‐2b) and 4 tumor‐derived nonsmall cell lung cancer (NSCLC) lines (A549, H358, H1299, H1703). Relative expression values are plotted on a logarithmic scale (log10). Data represent the mean ± SD of three independent biological experiments, each performed in technical triplicate (*n* = 3). (B) Effect of *PIK3R1* isoforms on cell proliferation rate in the lung cancer cell line, H358. The Alamar Blue cell proliferation assay was performed across different time intervals (0, 24, 48, and 72 h) following transient transfection using siRNA specific to p85α and p55α and the mock control (scrambled siRNA). Fluorescence intensity was measured at each time point, and the relative growth curve was plotted for the control (red), p85α (blue), and p55α (green). While knockdown with the p85α isoform significantly enhanced cell proliferation rate at 48 and 72 h, knockdown with the p55α isoform inhibited cell proliferation rate at 48 and 72 h compared to the control. The scale is indicated on the left side (O.D./Time points), and the error bars represent the standard deviation. Two‐way ANOVA, followed by Sidak's multiple comparison tests compared the differences in cell proliferation rates between the scrambled siRNA (control), p85α siRNA and p55α siRNA at different time points. Statistical significance is denoted by asterisks: ****P < 0.0001.

qRT‐PCR was performed on a panel of lung cancer cell lines to determine the expression levels of p85α and p55α (Fig. 7A). As compared to the cancer cell lines, p85α was consistently expressed at higher levels in normal cell lines, indicating p85α as a tumor suppressor. On the other hand, p55α was not expressed in normal cell lines; however, a moderate increase was observed in selected tumor lines, suggesting a potential oncogenic role in tumor biology. Based on the data, we selected the cancer cell line H358 (lung cancer). Using siRNA‐mediated knockdown, we evaluated changes in cellular proliferation (Fig. 8). Following successful downregulation in the H358 cell line (Fig. S7), we analyzed the proliferative ability of the transfected cell line in comparison with their scramble control using the Alamar Blue proliferation assay; our data revealed that depletion of the p55α isoform showed a significant decrease in cell proliferation (Fig. 8) as compared to the p85α isoform scramble control after 24 h. These *in vitro* studies provided further evidence supporting the opposing roles of p85α and p55α in cancer biology, consistent with our pan‐cancer and clinical data analyses.

Notably, in this study, we performed the BaseScope Assay for the simultaneous detection and spatial localization of the two RNA targets, the primary isoform p85α (labeled green) and the splicing variant p55α (labeled red) of *PIK3R1* at single‐cell resolution in LUSC tissue samples. Each set of panels (Fig. S8A–H,I–P) corresponds to a separate tissue sample. H&E staining (Panels A–B, I–J) shows the general morphology of the tissue, while panels C–E and K–M show the negative control using probes against housekeeping genes to demonstrate assay specificity and minimal background. The BaseScope duplex signal (Panels F and N) visualizes the expression of each isoform as distinct green or red dots within cells. Finally, panels G–H and O–P quantify these signals across different tissue regions, providing insights into the relative abundance and localization of each isoform. Data from the BaseScope™ Assay showed little to no signal in the samples (Fig. S8).

### 3.4 Correlation between the protein expression of the primary isoform of PIK3R1 (p85α) and its splicing variant (p55α) with target players of the PI3K/Akt pathway

To understand the underlying mechanisms underpinning p85α and p55α‐induced cancer, we analyzed expression of the key target players involved in the PI3K/Akt pathway (PIK3R1, PIK3R2, PIK3CA, p85α, p110a, Akt 1/2/3, Akt_pS473, Akt_pT308, mTOR, mTOR_pS2448, PTEN, and p53) in correlation to p85α and p55α expression (Fig. S9).

**Fig. 9:**
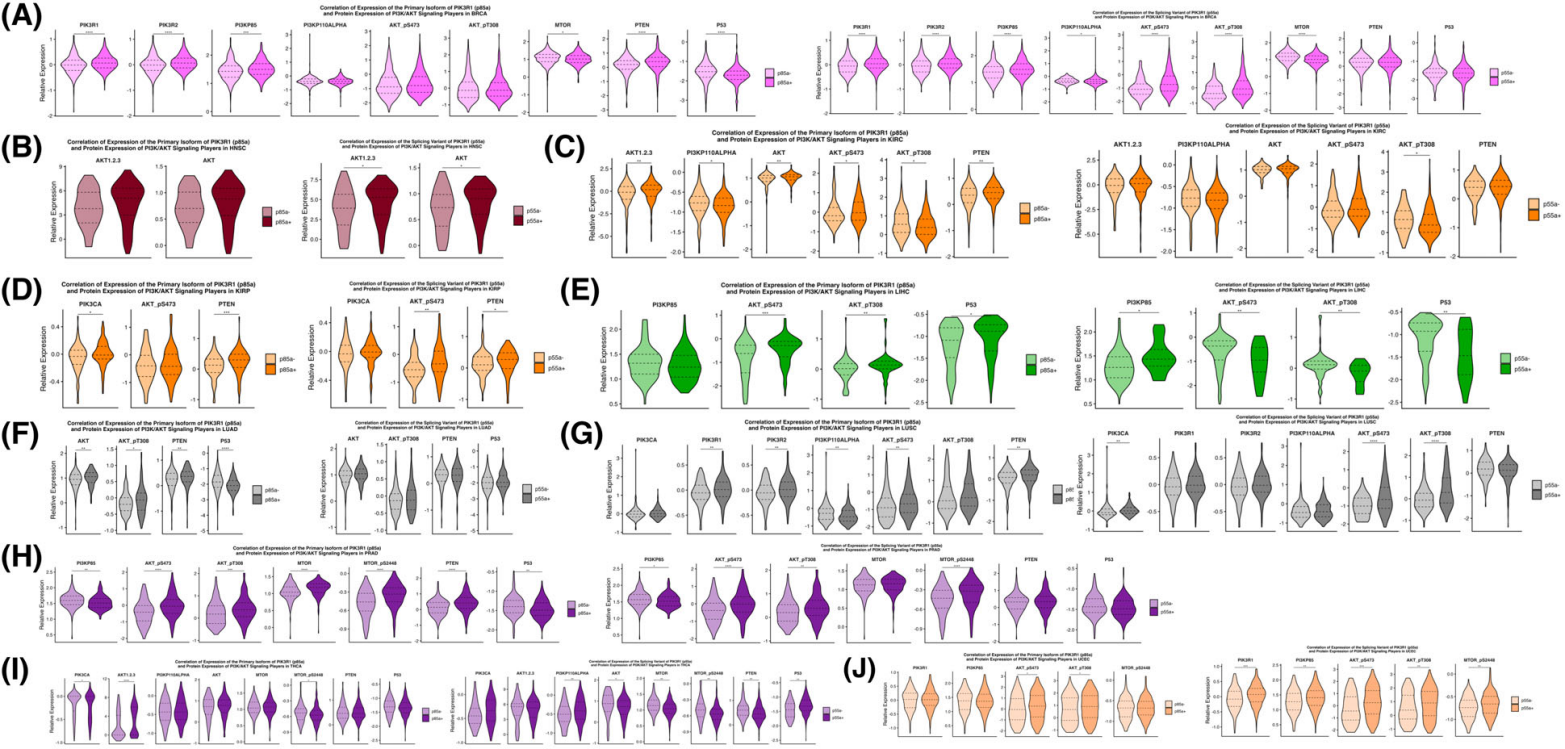
Correlation between the expression levels of the primary isoform of *PIK3R1* (p85α) and the splicing variant of *PIK3R1* (p55α) with target players of the PI3K/Akt pathway, across (A) BRCA, (B) HNSC, (C) KIRC, (D) KIRP, (E) LIHC, (F) LUAD, (G) LUSC, (H) PRAD, (I) THCA, and (J) UCEC. The figure displays violin plots illustrating the significant correlation between expression levels of the primary isoform of *PIK3R1*, p85α, and its splicing variant, p55α with target players of the PI3K/Akt pathway (PIK3CA, p85α, p110a, PIK3R1, Akt, Akt1/2/3, Akt_pS473, Akt_pT308, mTOR, mTOR_pS448, and PTEN) across the cancer types. Each violin represents a different target protein, showing the spread of p85α or p55α expression levels across samples, with the width indicating the density of the data points at different expression levels. One‐way ANOVA tests were performed to determine the significance of the differences in expression levels of each target protein based on the low and high expression of the primary isoform (p85α‐ and p85α+, respectively) as well as the splicing variant (p55α‐ and p55α+, respectively) of *PIK3R1*. Statistical significance is denoted by asterisks: *P < 0.05, **P < 0.01, ***P < 0.001, ****P < 0.0001. Both panels provide a visual representation of the correlation between the *PIK3R1* isoforms and the target proteins, with the median and interquartile ranges marked within each violin. Only statistically significant correlations are shown in the main figure to highlight the most relevant interactions. Complete correlation data for all proteins analyzed are provided in Fig. S8. BRCA, Breast invasive carcinoma; KIRC, Kidney renal clear cell carcinoma; KIRP, Kidney renal papillary cell carcinoma; LIHC, Liver hepatocellular carcinoma; LUAD, Lung adenocarcinoma; LUSC, Lung squamous cell carcinoma; PRAD, Prostate adenocarcinoma; THCA, Thyroid carcinoma; UCEC, Uterine Corpus Endometrial Carcinoma.

In the pan‐cancer analysis of the 11 types of human cancers, a significant negative correlation was observed between p85α and PIK3CA expression in BRCA (Fig. 9A), while no such correlations between p85α and PIK3CA expression were found in other cancer types (Fig. 9). Interestingly, in BRCA, p85α expression showed a positive correlation with PTEN levels (Fig. 9A). Likewise, while in KIRC, KIRP, and PRAD, a significant association was found between p85α and PTEN expression, no association was found in the other 7 types of human cancers (Fig. 9B,C,G, respectively). Furthermore, we also investigated the correlation of p85α with PIK3R2 and TP53, two additional key components of the PI3K/Akt pathway. Across all analyzed cancer types, no significant correlation was observed between p85α and PIK3R2 (Fig. 9). Notably, our data demonstrate statistically significant correlations between p85α expression and p53 protein levels in BRCA and LUAD (Fig. 9A,E), highlighting a potential functional relationship between p85α expression and p53 status.

With respect to total Akt levels, high p85α expression significantly correlated with total Akt only in THCA (Fig. 9H), whereas in PRAD, low p85α expression was associated with increased total Akt (Fig. 9G). A positive correlation between high p85α expression and Akt1/2/3 was detected in KIRC, PRAD, and THCA (Fig. 9B,G,H, respectively). We also analyzed the phosphorylation status of Akt (Akt_pS473 and Akt_pT308) (Fig. 9). Our data show that high p85α expression significantly correlates with Akt_pS473 in LIHC, LUSC, PRAD, and UCEC (Fig. 9D,F,G,I, respectively). However, contradictory results were observed for Akt_pT308; while in KIRC, low p85α expression was associated with Akt_pT308 (Fig. 9B), high p85α expression was found to correlate with Akt_pT308 in LIHC, PRAD, and UCEC (Fig. 9D,G,I, respectively).

Additionally, pan‐cancer analysis of the 11 types of human cancers also revealed significant associations with target players of the PI3K/Akt pathway (Fig. 9). A significant correlation was observed between p55α and p85α, p110α, as well as PIK3R1/2 expression in BRCA, LIHC, PRAD, THCA, and UCEC (Fig. 9A). A significant correlation was observed between p55α and PIK3CA expression in LUSC (Fig. 9A), while no such correlations between p55α and PIK3CA expression were found in other cancer types (Fig. 9). In KIRP, while a negative correlation was found between p55α and PTEN expression, in THCA, a positive correlation was reported. However, contradictory results were observed for p53; while in LIHC, high p55α expression was associated with low p53 (Fig. 9B), high p55α expression was found to correlate with p53 levels in THCA (Fig. 9D,G,I, respectively). Furthermore, no such correlations between p55α and p53 expression were found in other cancer types.

With respect to total Akt levels, high p55α expression significantly correlated with total Akt and Akt1/2/3 only in HNSC (Fig. 9H), whereas in PRAD, high p55α expression was associated with decreased total Akt (Fig. 9G). Analysis of phosphorylation status of Akt (Akt_pS473 and Akt_pT308) revealed high p55α expression was significantly associated with Akt_pS473 and Akt_pT308 in BRCA, LUSC, PRAD, and UCEC (Fig. 9I), suggesting a potential link to PI3K activation via Akt phosphorylation. Likewise, in KIRP, high p55α expression was significantly associated with Akt_pS473. With respect to mTOR expression, in BRCA and PRAD, high p55α expression was associated with loss of mTOR. Contradictory results were observed for phosphorylation of mTOR (mTOR_pS2448), in PRAD and UCEC, while high p55α expression induced mTOR phosphorylation, in THCA high p55α expression inhibited mTOR phosphorylation.

### 3.5 Mutational characteristics of PIK3R1 in different tumors of TCGA

We next analyzed genetic variants in both the primary isoform of *PIK3R1* (p85α) and its splicing variant (p55α). In the primary isoform of *PIK3R1*, p85α, a total of 2893 variants were reported; however, we found no mutation in the coding region of the splicing variant, p55α (Table 1).

**Table 1:** Genetic variants identified in the primary isoform of *PIK3R1* (p85α) and its splicing variant (p55α).

p85α 2893 variants	p55α 445 variants	rsID	Type and length	Transcript consequence	Clinical significance	Disease
5′ UTR variants (428)	None	rs143667799	SNV, 1 bp	c.‐354G>C	Likely benign	Not specified
		rs138814985	Microsatellite, 5 bp	c.‐212‐208dup	Benign	Not specified
		rs2888323	SNV, 1 bp	c.‐207A>G	Benign	
Intron variants (2372)	Intron variants (436)	rs1747126586	SNV, 1 bp	c.‐917‐4792G>A	Uncertain significance	Immunodeficiency 36 SHORT syndrome
		rs557298855	SNV, 1 bp	c.‐917‐3348G>A	Likely benign	Not provided
		rs200046946	SNV, 1 bp	c.‐916 + 9 T>G	Benign	Agammaglobulinemia 7, autosomal recessive Immunodeficiency 36 SHORT syndrome
		rs3730086	SNV, 1 bp	c.916 + 217G>A	Benign	Not provided
		rs1862162	SNV, 1 bp	c.‐917‐3329A>G	Benign	Not provided
		rs3730087	SNV, 1 bp	c.‐917‐93C>T	Benign	Not provided
		rs3730088	SNV, 1 bp	c.‐917‐87A>G	Benign	Not provided
Splice region variants (11)	Splice region variants (9)	rs566631081	SNV, 1 bp	c.‐917‐3C>T	Likely benign	Agammaglobulinemia 7, autosomal recessive Immunodeficiency 36 SHORT syndrome
Splice donor variants (4)	None	N/A	SNV, 1 bp	c.‐916 + 2 T>C (Donor splice site in intron 7 of the *PIK3R1* gene)	Likely pathogenic	Agammaglobulinemia 7, autosomal recessive Immunodeficiency 36 SHORT syndrome
Missense variants (51)	None	rs144312303	SNV, 1 bp	c.18G>T	Conflicting pathogenicity	Agammaglobulinemia 7, autosomal recessive Immunodeficiency 36 SHORT syndrome
Start_Lost (5)	None	N/A	N/A	N/A	N/A	N/A
Stop_Gained (2)	None	N/A	N/A	N/A	N/A	N/A
Synonymous variants (16)	None	N/A	N/A	N/A	N/A	N/A

Of the intronic variants identified, only 2 of the variants (rs3730087 and rs3730088) were common between p85α and p55α. In addition, of the splice region variants noted, only rs566631081 was common between p85α and p55α.

Notably, while all the genetic variants did not affect the protein consequence of *PIK3R1*, only the missense variant, rs144312303, alters the protein consequence at position 6, where the amino acid tryptophan is replaced by cysteine (p.Trp6Cys).

## 4 Discussion

PI3K, a heterodimer composed of the regulatory (p85α, p85b, p50α, p55α, or p55g) and the catalytic (p110a, p110b, or p110d) subunits, plays a vital role in regulating the PI3K/Akt pathway which is frequently deregulated during carcinogenesis [1]. Among these, *PIK3R1* encodes the p85 regulatory subunit and its splicing variant, p55α, regulates RTK association with the p110 catalytic subunit [5]. Several *in vitro* and *in vivo* studies have demonstrated the role of the full‐length p85α isoform in carcinogenesis [25, 30, 43, 44, 45, 46]. However, while studies have highlighted the role of the splicing variant p55α in insulin signaling [47, 48, 49], studies in cancer are nascent. To our knowledge, this is the first study to provide a comprehensive pan‐cancer analysis of the primary isoform (p85α) and the splicing variant (p55α) of *PIK3R1*, demonstrating their opposing roles in tumorigenesis.

Concordant with other studies, we observed a significant downregulation of p85α in tumors relative to normal tissues, suggesting a tumor‐suppressive role in cancer. Loss of p85α has previously been shown to trigger the PI3K pathway through reduced inhibition of p110α, leading to enhanced Akt phosphorylation and downstream signaling [50, 51]. In agreement with findings by Thorpe et al. [46], our analysis confirmed reduced p85α levels to correlate with activation of the PI3K/Akt pathway. Likewise, in hepatocellular carcinoma, loss of *PIK3R1* expression activated the PI3K pathway, leading to the development of hepatocellular carcinoma with metastasis to the lungs [51, 52]. Our findings are also supported by a study by Liu et al. (2022) [27], who reported loss of *PIK3R1* expression to activate the PI3K/Akt pathway. However, while the study analyzed total *PIK3R1* expression, our study distinguishes between the isoforms and demonstrates that p85α loss specifically contributes to pathway activation and poor prognosis. Our data further reinforce this tumor‐suppressive role, with lower p85α expression correlating with poor overall survival in patients with BRCA, KIRC, and LUAD. Low p85α expression was also associated with poor prognosis in BRCA, HNSC, KIRC, and PRAD, as previously reported [27, 52]. On the other hand, for the first time, we reported the correlation between high p55α expression and patient outcome; while high p55α expression was significantly associated with poor overall survival in KICH, high p55α expression was associated with poor prognosis in KIRC. Therefore, the expression pattern and prognostic value of p55α in kidney cancer deserve further exploration based on larger sample sizes and clinical data. These observations of pan‐cancer analysis further confirmed that p85α, as well as p55α, may have opposing roles in cancers.

We also examined racial disparities, given the known differences in cancer outcomes between AA and EA populations [53]. Factors such as genetic mutations, comorbidities, delayed diagnosis, and access to care contribute to these disparities [54, 55, 56, 57]. The aggressive nature is driven by differences in molecular pathways, including PI3K [53], as well as due to certain alternative splicing events as noted in *PIK3CD*, *FGFR3*, *RASGRP2*, and *TSC2* [58], suggesting AA tumors have unique molecular characteristics. In this context, we further delved into the expression analysis of p85α and p55α in the different racial ethnic groups (AA and EA) along with correlation with patient outcome. While in certain cancer types, a significant difference in p85α expression was observed between the racial groups, there was no significant difference in p55α expression between the racial groups. Moreover, correlation of the isoforms and patient outcome revealed distinct outcomes in AAs and EAs, suggesting distinct regulatory dynamics that may be influenced by lower mutation rates in *PIK3R1*, *PIK3CA*, and *PTEN* [54, 59]. The differential mutation frequency can result in distinct patterns in PI3K pathway activation and potentially influence the expression of *PIK3R1* isoforms.

To further explore the underlying mechanisms, we analyzed the correlation of p85α and p55α expression with the target players involved in the PI3K/Akt pathway. Our data indicate loss of p85α to activate the PI3K/Akt pathway as demonstrated by enhanced total Akt, phosphorylation of Akt at Thr308 residue, and mTOR phosphorylation and Ser2448 residue. This is in line with previous studies showing that loss of p85α in mouse liver leads to increased Akt activation and PTEN loss [60]. Furthermore, we observed positive correlations between p85α and *PTEN* expression in BRCA, KIRC, KIRP, and PRAD, suggesting that p85α stabilizes *PTEN* and has a positive regulatory effect on the function of *PTEN* [51, 61]. These findings are further supported by Cizkova et al. (2013) [52] who demonstrated that reduced *PIK3R1* expression in BRCA is associated with *PTEN* loss and increased Akt activation, reinforcing the role of p85α in maintaining *PTEN* stability and restraining PI3K/Akt signaling. However, in the other cancer types (HNSC, KICH, LICH, LUAD, LUSC, THCA, and UCEC), this correlation was not significant, thus suggesting loss of *PTEN* as a secondary event rather than a primary driver of the transformation due to reduced p85α levels. On the other hand, loss of *PTEN* correlates with wild‐type *PIK3CA* and loss of p85α; in line with previous findings, we noted a significant correlation between loss of p85α and wild‐type *PIK3CA* in BRCA [52]. We further found significant correlations between p85α expression and p53 protein levels in specific tumor types, including BRCA and LUAD, aligning with the established crosstalk between the PI3K/Akt and p53 pathways. PI3K/Akt signaling can negatively regulate p53 through the MDM2‐mediated degradation, while p53 in turn can suppress PI3K/Akt signaling by transcriptionally activating *PTEN*, a negative regulator of PI3K [62, 63]. Additionally, we evaluated correlations with *PIK3R2*. Notably, our analysis revealed no significant correlation between the expression of p85α /p55α and PIK3R2 protein levels across the cancer types analyzed, suggesting that, despite *PIK3R2* being a known regulatory subunit of class IA PI3Ks, its expression may be independently regulated or functionally distinct from p85α/p55α. These findings are consistent with previous reports indicating that p85β functions independently of p85α and may possess distinct, potentially oncogenic roles [6, 64].

Studies have reported mutations in *PIK3R1* in different human cancers, which activate the PI3K pathway and stimulate downstream AKT signaling, potentially inducing carcinogenesis [25, 29, 30, 31]. In this study, we reported genetic variants of *PIK3R1*. Somatic mutations in *PIK3R1* have been reported in different human cancers [52, 61]. In the nSH2 and iSH2 domains of p85α, point mutations trigger the PI3K signaling pathway, leading to the onset and progression of glioblastoma [65]. Although the underlying mechanism of p85α transformation is nascent, cancer‐associated mutations in *PIK3R1* are reported to result in premature truncation of the p85α protein [45, 66]. Truncated p85α mutants lack the C‐terminal SH2 domain, plausibly losing their ability to bind to IRS1 when forming p85 homodimers [45, 46]. However, on the other hand, during the hemizygous loss of p85α, the remaining full‐length p85α may preferentially bind to p110α, facilitating binding to *IRS1* [45, 46]. This indicates that, similar to p85α reduced levels, truncated p85α mutations can potentially impact PI3K signaling by releasing binding sites on activated RTKs for signaling‐competent p85α‐p110 heterodimers [45, 46]. Furthermore, Thorpe and colleagues [46] further used *in vivo* models and demonstrated that in cancers with reduced p85α levels, pan‐ and p110α‐specific inhibitors (BYL‐719) inhibited the growth of transplanted NIC tumors lacking *PIK3R1*, thus suggesting using pan‐ and p110α‐specific inhibitors in tumors lacking p85α levels.

This pan‐cancer analysis of the primary isoform (p85α) and the splicing variant (p55α) of *PIK3R1* highlights the significant impact of their aberrant expression in tumorigenesis and patient outcome, underscoring the necessity for further research. However, the study has several limitations. Despite utilizing various databases to explore p85α and p55α functions in a pan‐cancer context through a bioinformatics approach, the original data, primarily sourced from the TCGA database, may differ in collection and processing methods across different databases, potentially introducing systemic bias. Moreover, despite growing recognition of racial and ethnic disparities in cancer outcomes, Native American populations remain significantly under‐represented in genomic studies, further limiting our ability to assess their specific disease characteristics and responses to therapy. Although eleven patients identified as Native American were included in our dataset, we did not perform subgroup analysis or draw conclusions due to the insufficient sample size; this highlights the need for future studies with greater representation of under‐represented populations. On the other hand, despite observing upregulated levels of p55α in tumor samples, the BaseScope™ assay did not yield detectable signals for this isoform in our samples. However, the BaseScope assay is a semi‐quantitative approach with a detection limit [67]. One of the reasons for the lack of data could be either due to the sensitivity of the assay to detect specific isoforms or due to the degradation of the Fast Red colorimetric dyes [68, 69], thus suggesting that the assay's detection threshold may not be sufficient for certain isoforms. Future analysis should focus on optimizing the assay parameters, such as probe design and hybridization conditions, to enhance detection capabilities.

In addition to utilizing online databases and performing BaseScope™ Assay, we did not perform any functional *in vitro* or *in vivo* experiments. Thus, further studies focusing on the underlying cellular and molecular mechanisms of p85α and p55α can help in understanding their role in underpinning cancer onset and progression. The data obtained underscores the critical need for targeted therapies that consider race‐specific molecular differences, particularly to enhance outcomes for AA patients. By investigating the impact of mutations in *PIK3R1*, *PIK3CA*, and *PTEN* on p85α and p55α to drive cancer progression, research can aim to offer deeper insights into how these genetic changes can be utilized for more personalized treatment strategies. This further warrants the need to develop customized therapeutic approaches across various racial groups. Moreover, isoform‐specific PI3K inhibitors could be effective in treating cancers marked by the loss of p85α or an increase in p55α expression.

## 5 Conclusion

In conclusion, our comprehensive pan‐cancer analysis of the primary isoform (p85α) and the splicing variant (p55α) of *PIK3R1* demonstrated distinct expression patterns and significant associations with various clinical parameters across various tumor types. Specifically, in tumors, while p85α exhibits a tumor‐suppressive role, p55α acts as an oncogene in comparison to normal samples. Both isoforms were closely associated with clinical outcomes, as well as with key players of the PI3K/Akt pathway. These findings greatly contribute to our understanding of the distinct and overlapping roles of p85α and p55α in tumorigenesis, and further work can aid in developing these isoforms as potential biomarkers and therapeutic targets in cancer.

## Conflict of interest

The authors declare that no conflict of interest that could be perceived as prejudicing the impartiality of the research reported.

## Author contributions

Conceptualization: DAG; methodology: IG, YS, and AS; formal analysis: IG, YS, and AS; data curation: IG, YS, AS, JCP, MN, TG, DZK, JG, AH, and AA; writing—original draft preparation: IG; writing—review and editing: YS, JCP, and DAG; funding acquisition: DAG.

## Supporting information


**Fig. S1.** Transcriptional Expression Analysis of the (A) Primary Isoform of *PIK3R1* (p85α) and (B) Its Splicing Variant (p55α).

**Fig. S2.** Correlation between the Expression Levels of the Primary Isoform of *PIK3R1* (p85α) and Splicing Variant of *PIK3R1* (p55α) with Overall Survival (OS).

**Fig. S3.** Correlation between the Expression Levels of the Primary Isoform of *PIK3R1* (p85α) and Splicing Variant of *PIK3R1* (p55α) with Overall Survival (OS) based on Racial Disparity, across (A) BRCA, (B) KICH, (C) KIRP, (D) LIHC, (E) PRAD, (F) THCA and (G) UCEC.

**Fig. S4.** Correlation between the Expression Levels of the Primary Isoform of *PIK3R1* (p85α) and Its Splicing Variant (p55α) with Progression‐Free Interval (PFI).

**Fig. S5.** Correlation between the Expression Levels of the Primary Isoform of *PIK3R1* (p85α) and Its Splicing Variant (p55α) with Progression‐Free Interval (PFI) based on Racial Disparity, across (A) KICH, (B) KIRP, (C) LIHC, (D) LUAD, (E) LUSC, (F) PRAD, (G) THCA and (H) UCEC.

**Fig. S6.** Multivariate Cox Analysis identifying factors affecting Progression Free Interval.

**Fig. S7.** Relative expression of *PIK3R1* isoforms (p85α and p55α) after transient knockdown using siRNA specific to p85α and p55α for cell proliferation assay.

**Fig. S8.** BaseScope Duplex Detection and Quantification of the Primary Isoform of *PIK3R1* (p85α) and Splicing Variant of *PIK3R1* (p55α).

**Fig. S9.** Correlation between the Expression Levels of the Primary Isoform of *PIK3R1* (p85α) and the Splicing Variant of *PIK3R1* (p55α) with Target Players of the PI3K/Akt Pathway, across the 11 TCGA cancer types including (A) BRCA, (B) HNSC (C) KICH, (D) KIRC, (E) KIRP, (F) LIHC, (G) LUAD, (H) LUSC, (I) PRAD, (J) THCA and (K) UCEC.

**Table S1.** List of cell lines used. The table includes the list of lung cancer cell lines used in the study.

## Data Availability

The data underlying this article are available in the article and the Supporting Information.
