# Supplementary figures and images for: Correlation of the differential expression of PIK3R1 and its spliced variant, p55α, in pan‐cancer
Source: Mol Oncol. 2026 Jan 20;20(5):1299–322. doi: 10.1002/1878-0261.70205 (PMC13155144; doi:10.1002/1878-0261.70205)

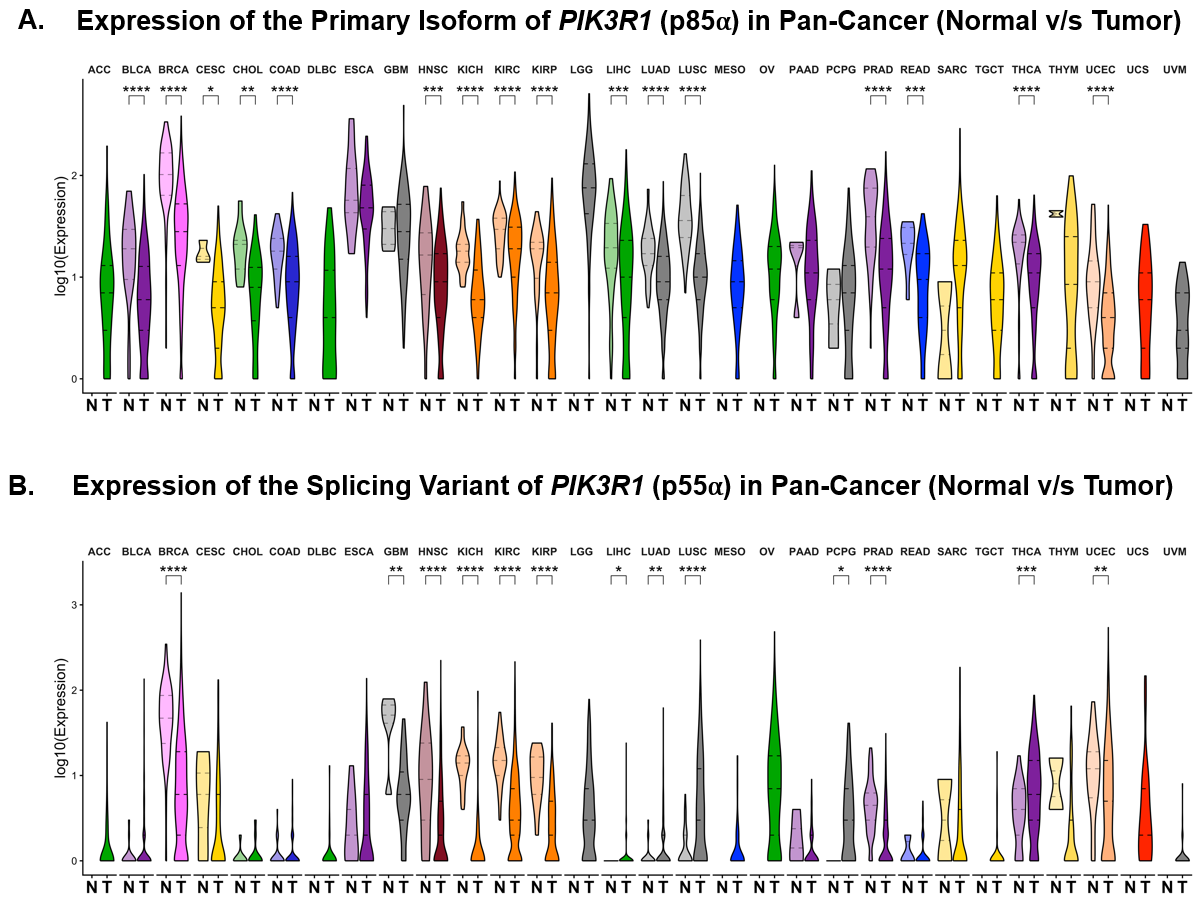

Supplement: Supplementary file 1 — Fig. S1. Transcriptional Expression Analysis of the (A) Primary Isoform of PIK3R1 (p85α) and (B) Its Splicing Variant (p55α). [file MOL2-20-1299-s002.png]

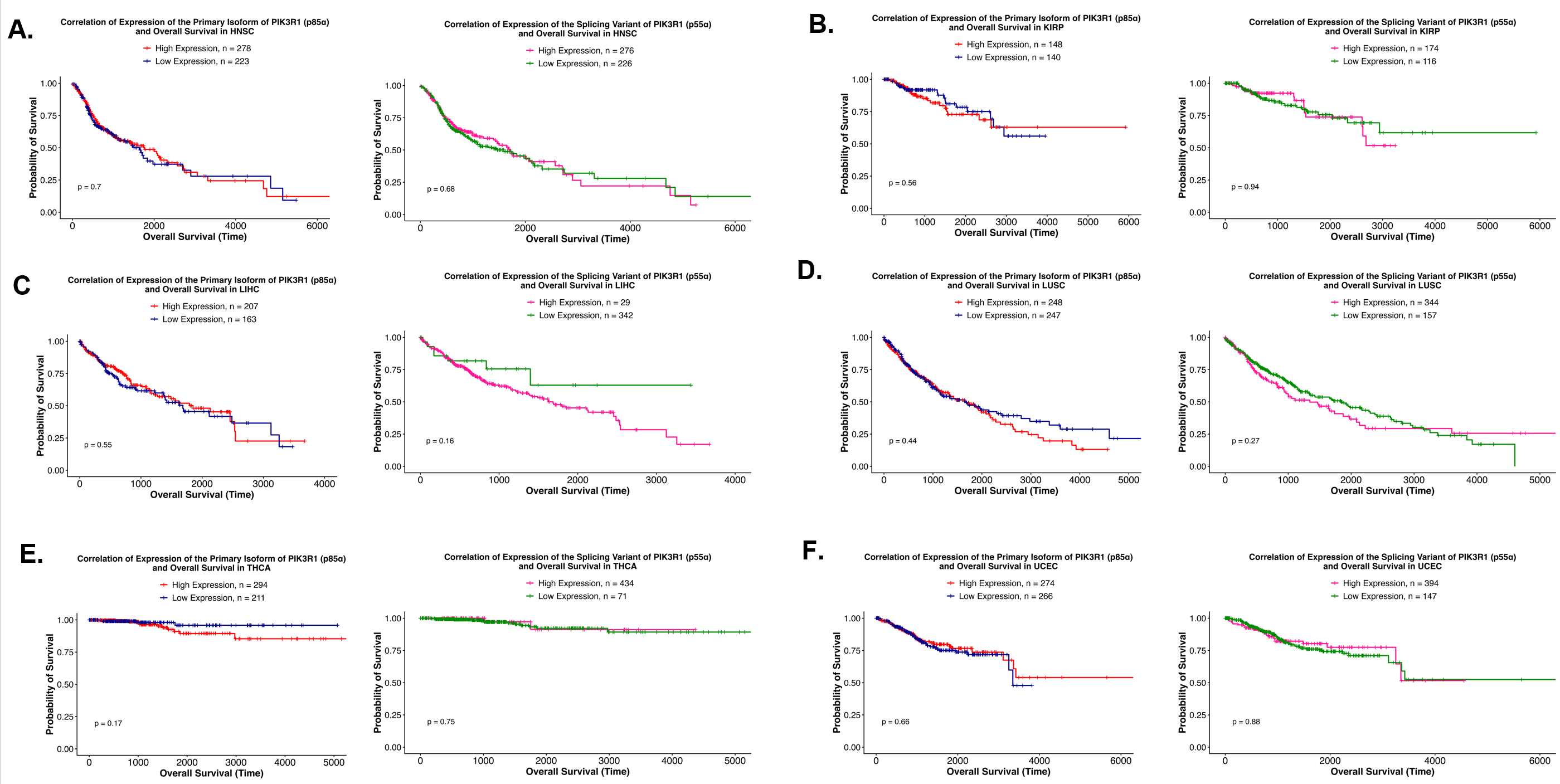

Supplement: Supplementary file 2 — Fig. S2. Correlation between the Expression Levels of the Primary Isoform of PIK3R1 (p85α) and Splicing Variant of PIK3R1 (p55α) with Overall Survival (OS). [file MOL2-20-1299-s008.png]

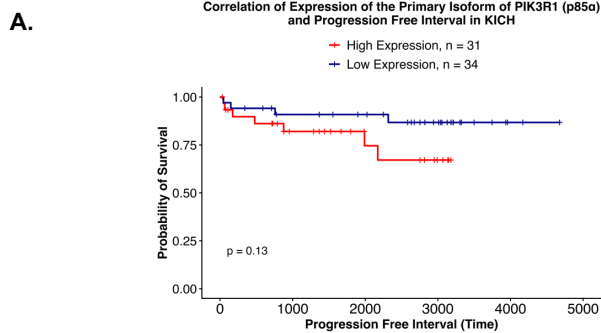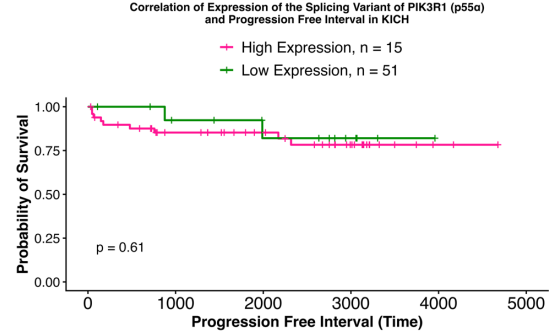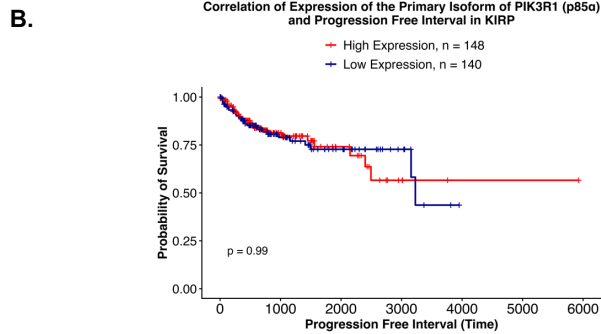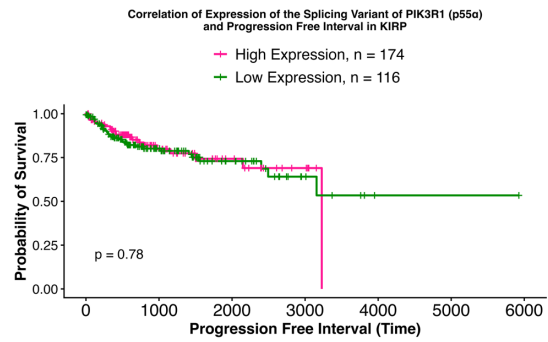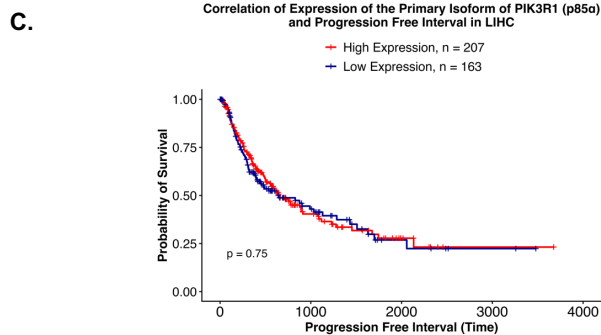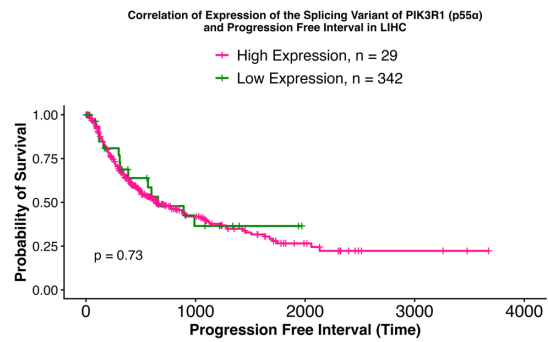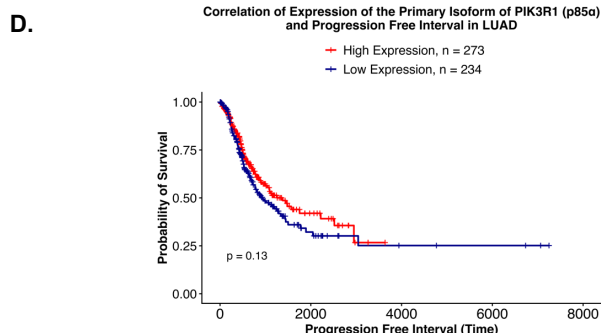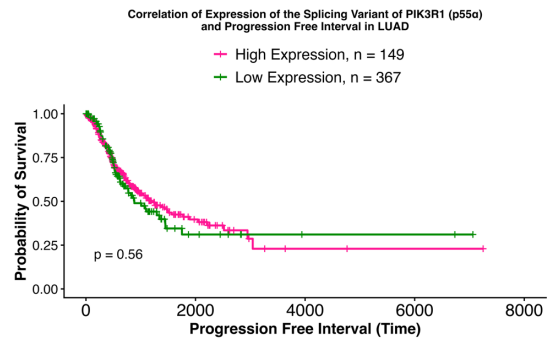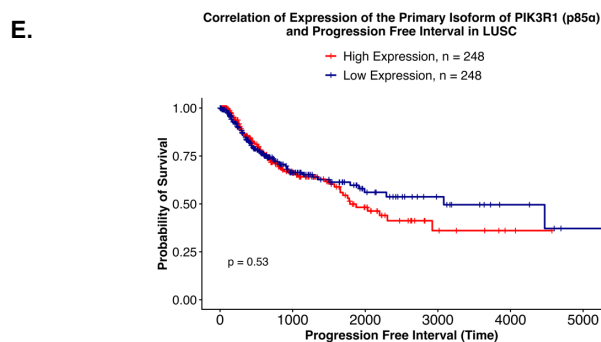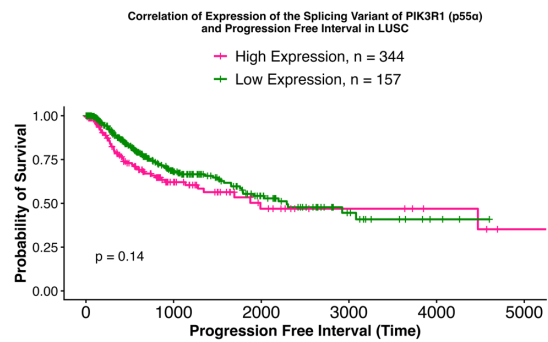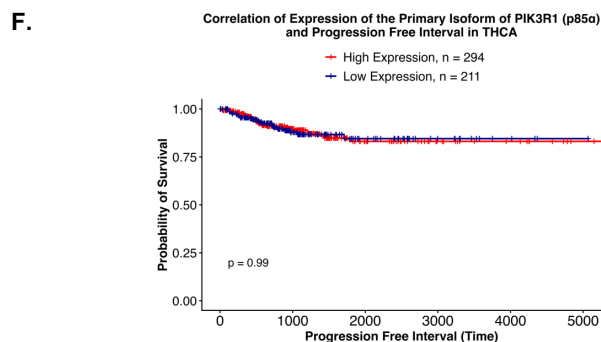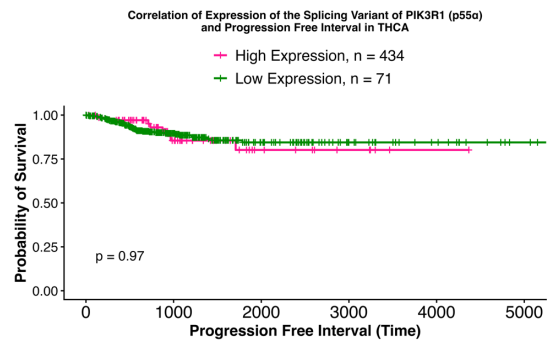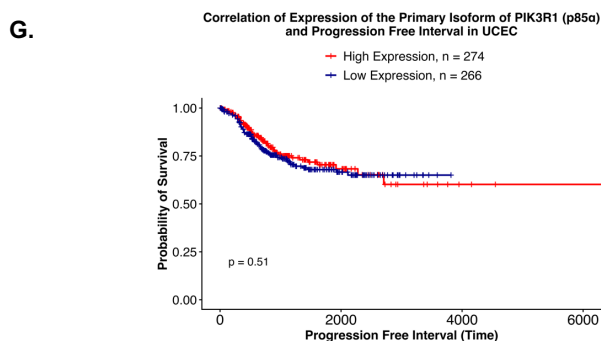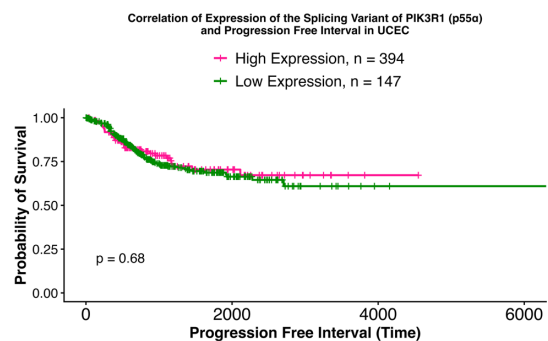

Supplement: Supplementary file 4 — Fig. S4. Correlation between the Expression Levels of the Primary Isoform of PIK3R1 (p85α) and Its Splicing Variant (p55α) with Progression‐Free Interval (PFI). [file MOL2-20-1299-s003.pdf]

**A.**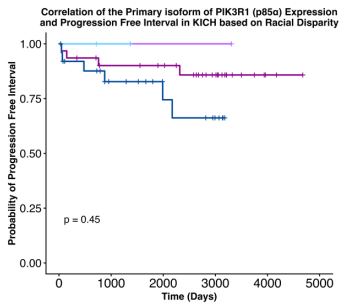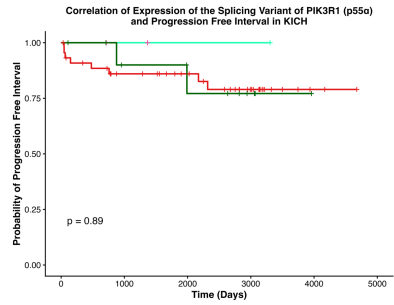**B.**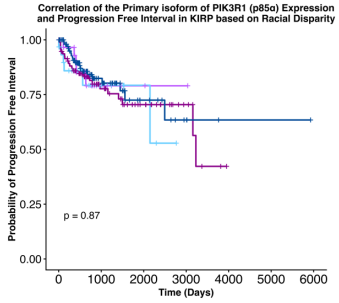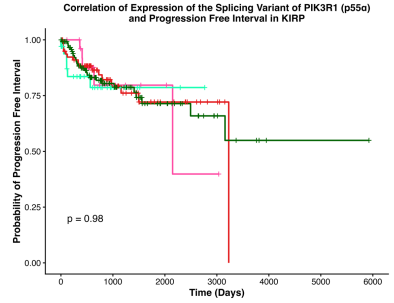**C.**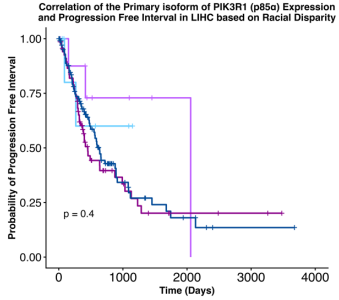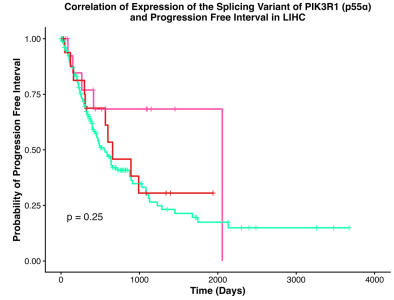**D.**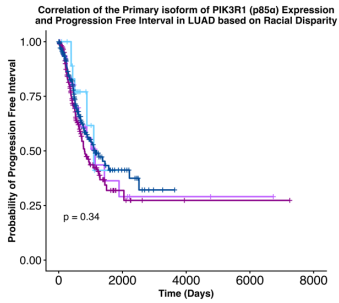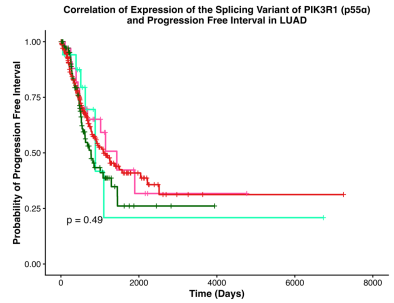**E.**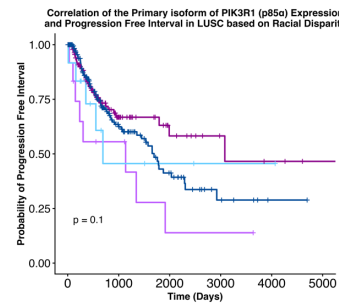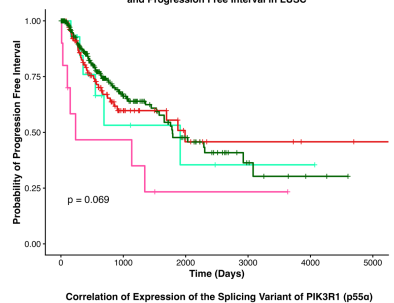**F.**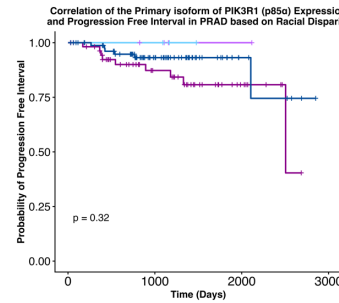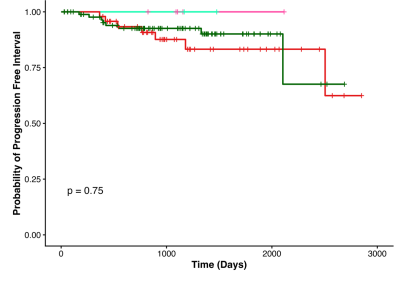**G.**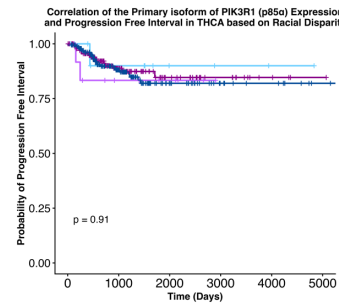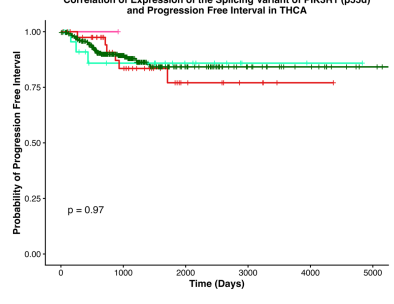**H.**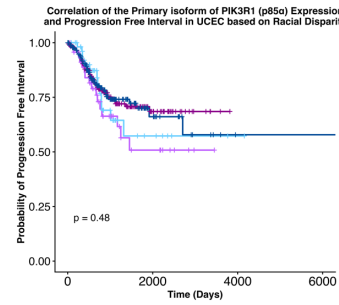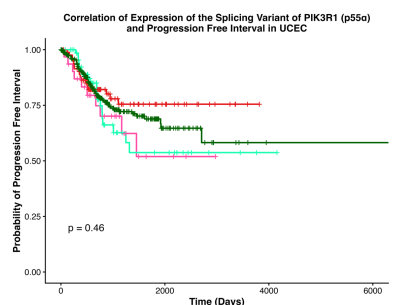

Supplement: Supplementary file 5 — Fig. S5. Correlation between the Expression Levels of the Primary Isoform of PIK3R1 (p85α) and Its Splicing Variant (p55α) with Progression‐Free Interval (PFI) based on Racial Disparity, across (A) KICH, (B) KIRP, (C) LIHC, (D) LUAD, (E) LUSC, (F) PRAD, (G) THCA and (H) UCEC. [file MOL2-20-1299-s010.pdf]

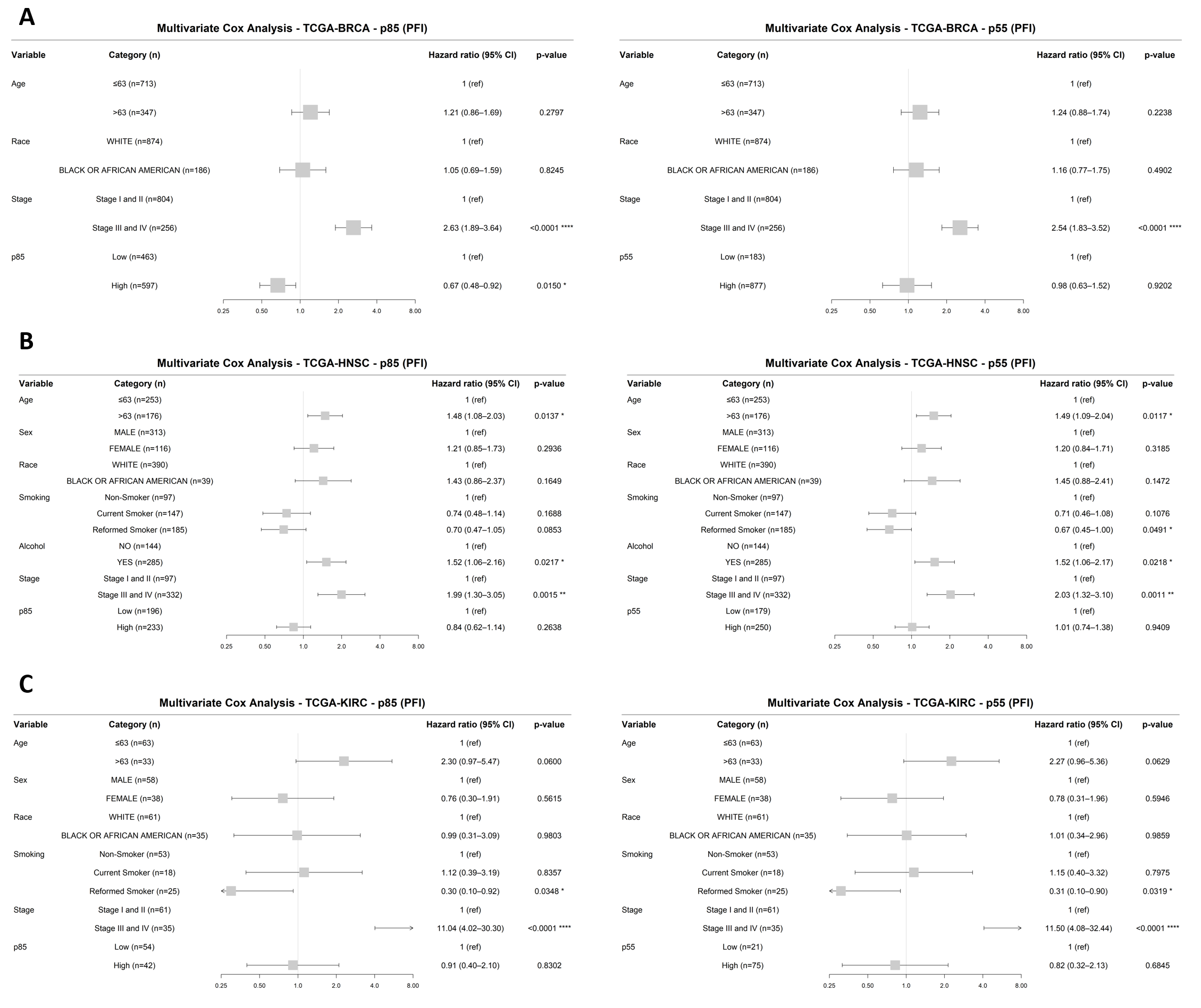

Supplement: Supplementary file 6 — Fig. S6. Multivariate Cox Analysis identifying factors affecting Progression Free Interval. [file MOL2-20-1299-s001.png]

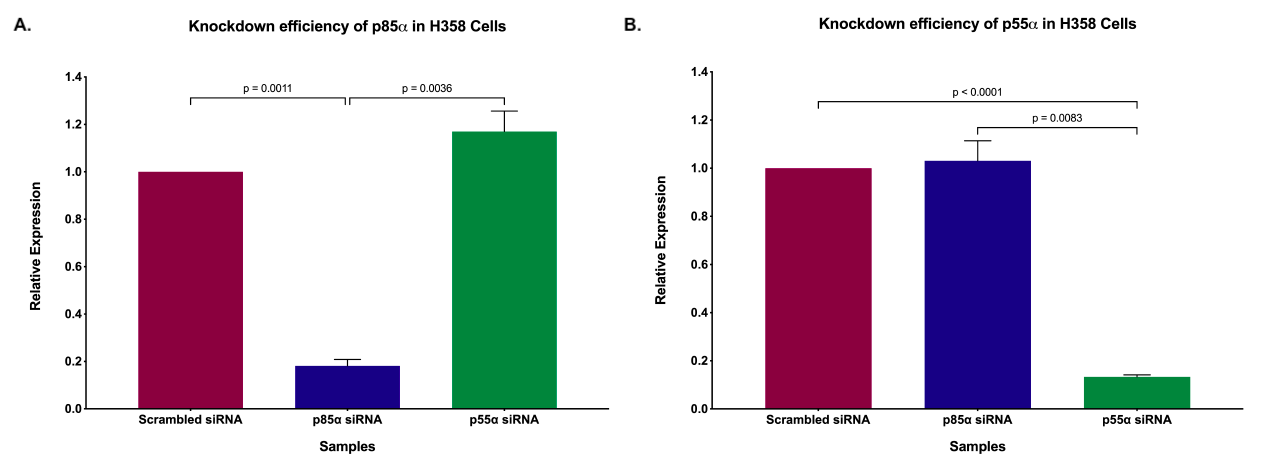

Supplement: Supplementary file 7 — Fig. S7. Relative expression of PIK3R1 isoforms (p85α and p55α) after transient knockdown using siRNA specific to p85α and p55α for cell proliferation assay. [file MOL2-20-1299-s005.png]

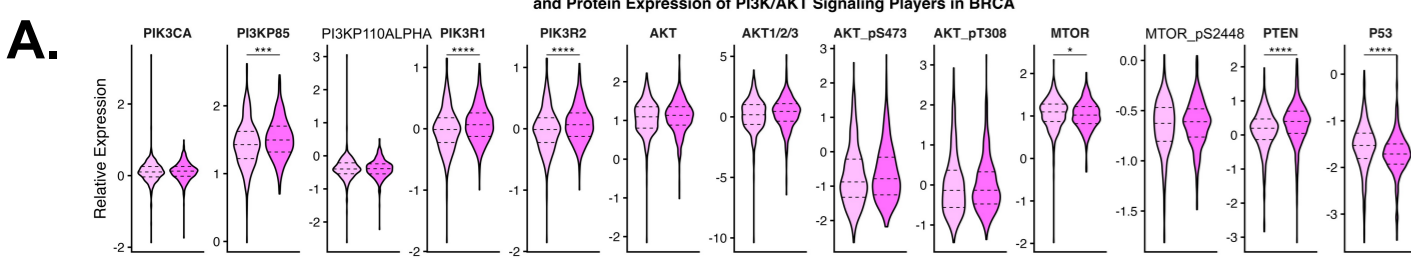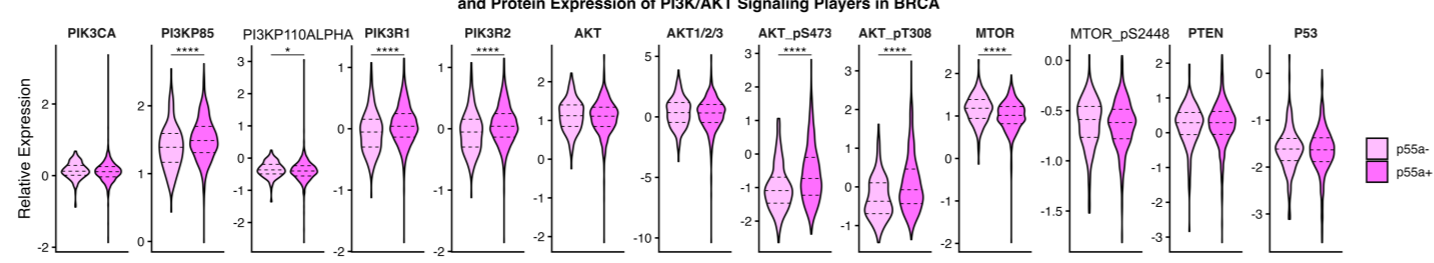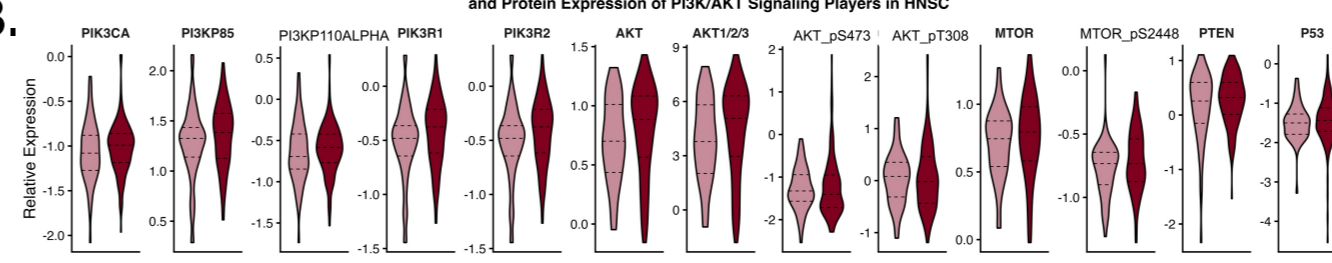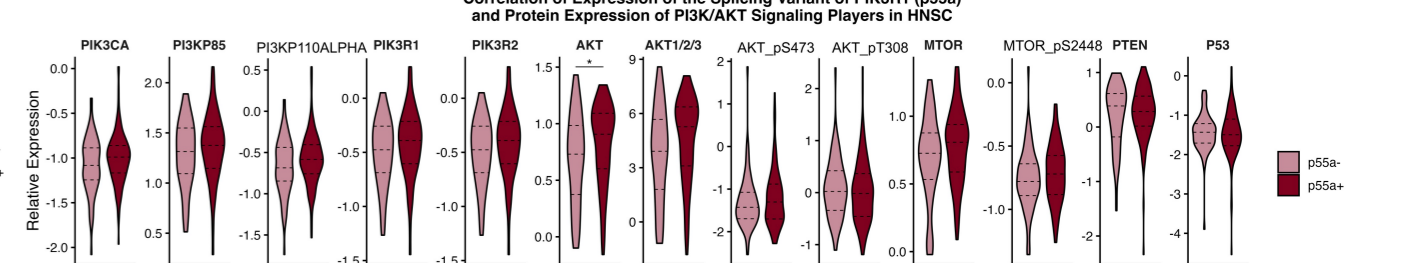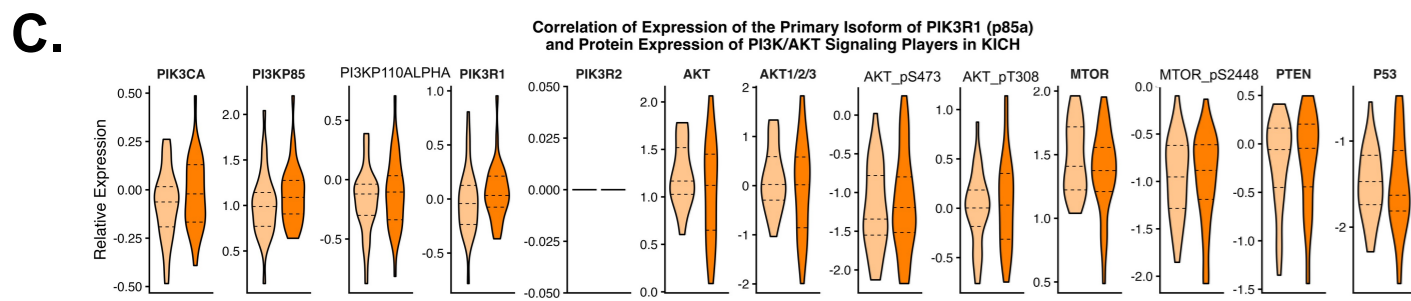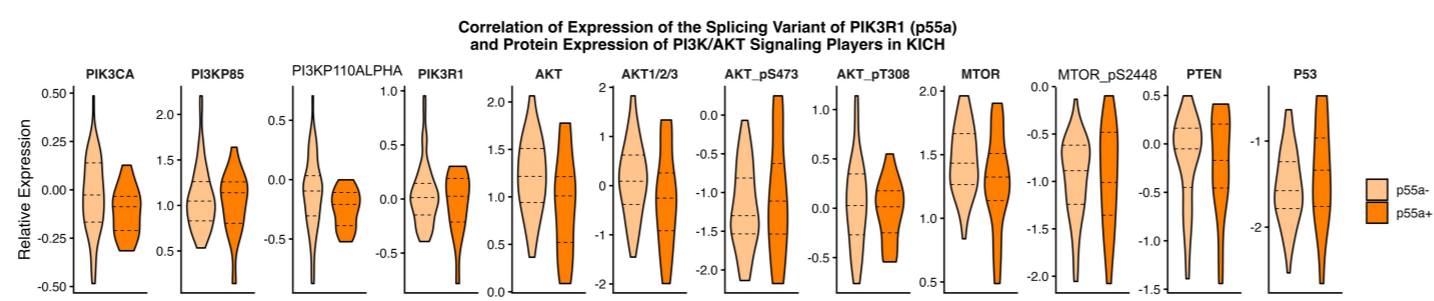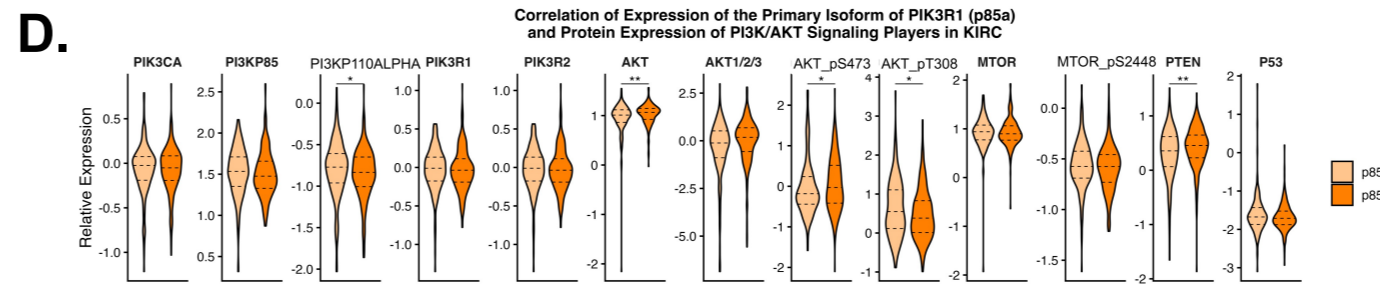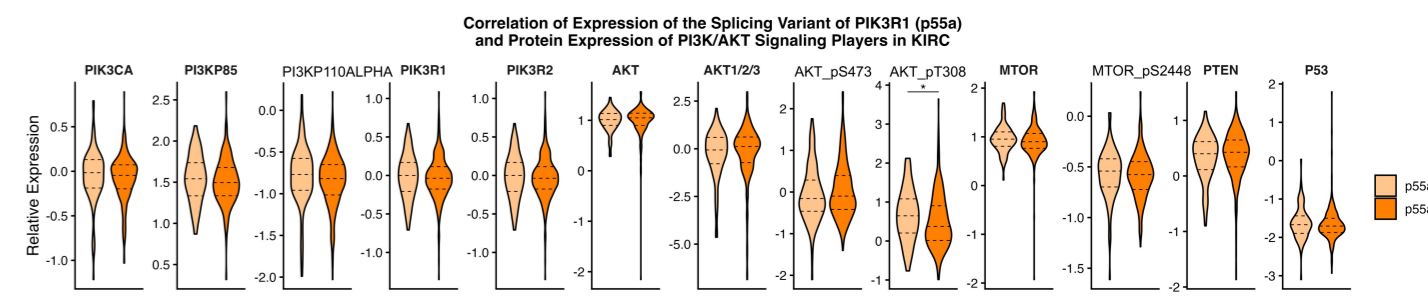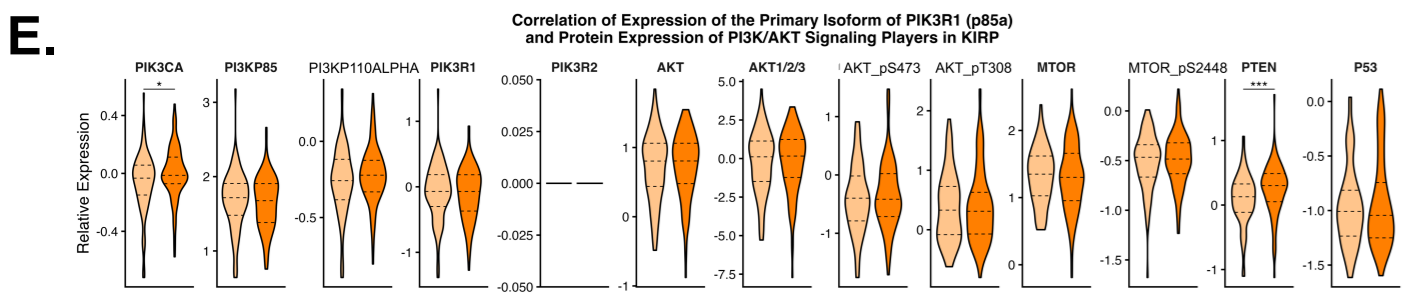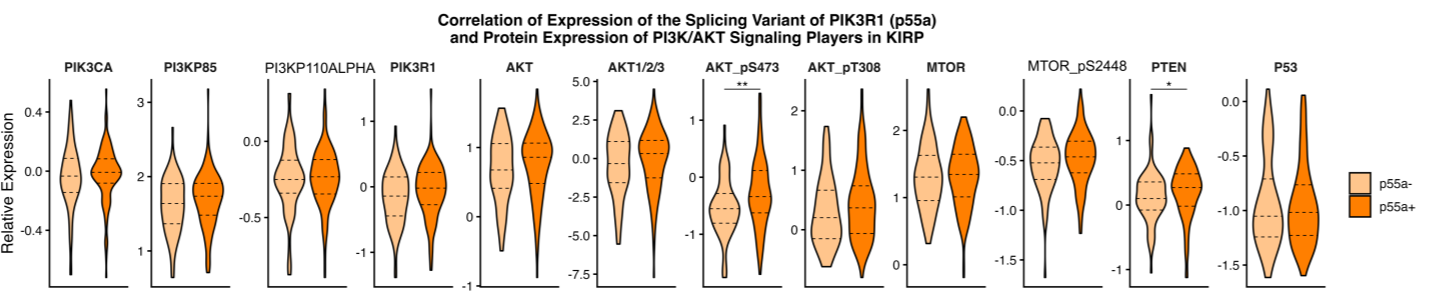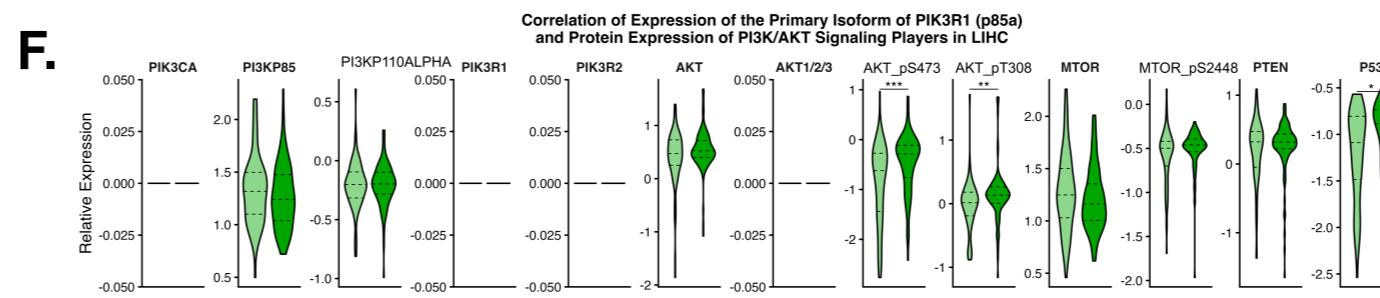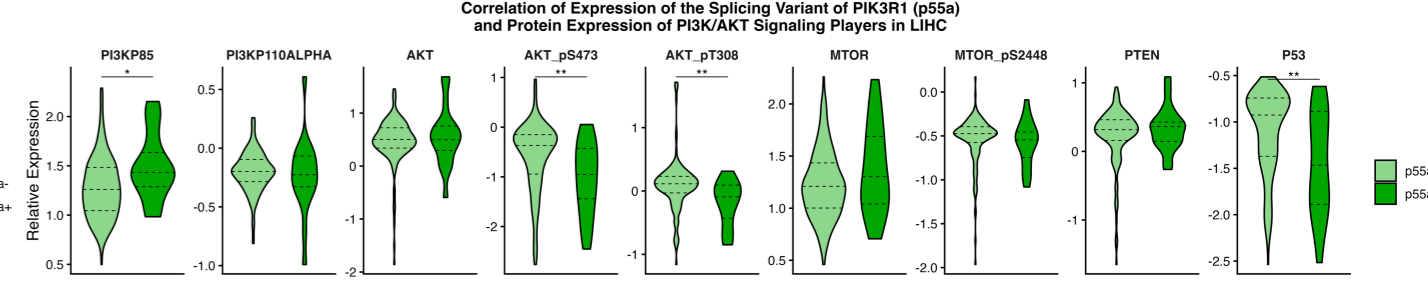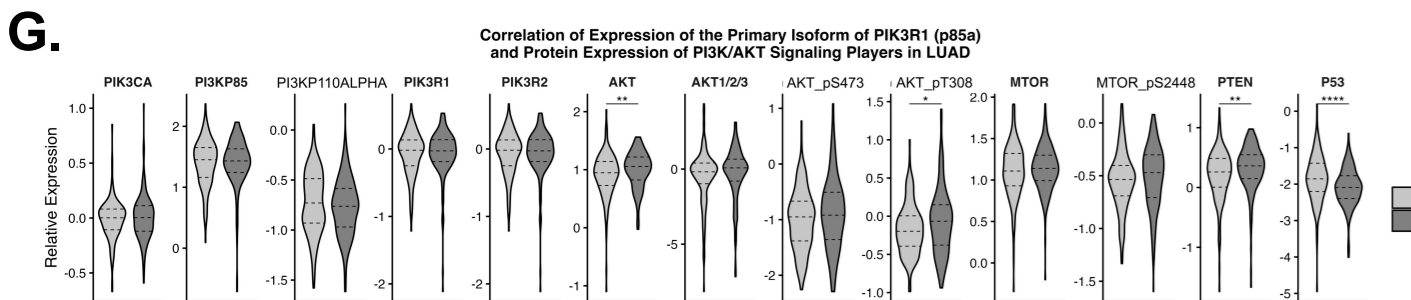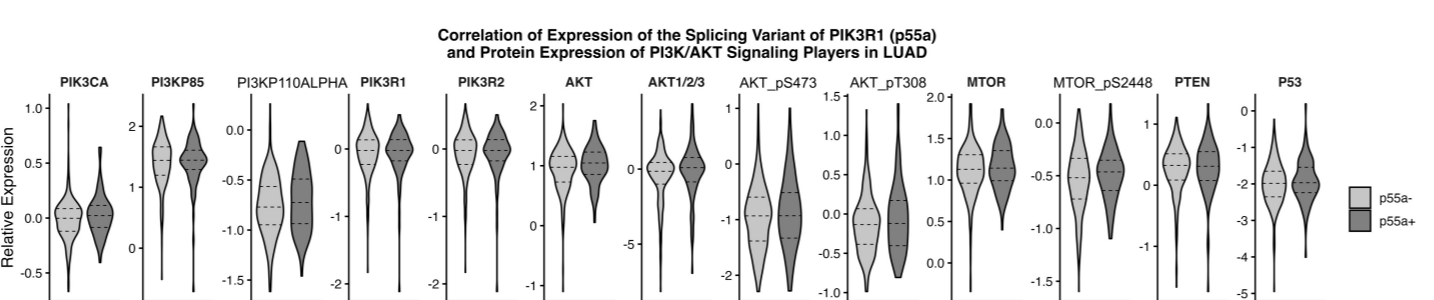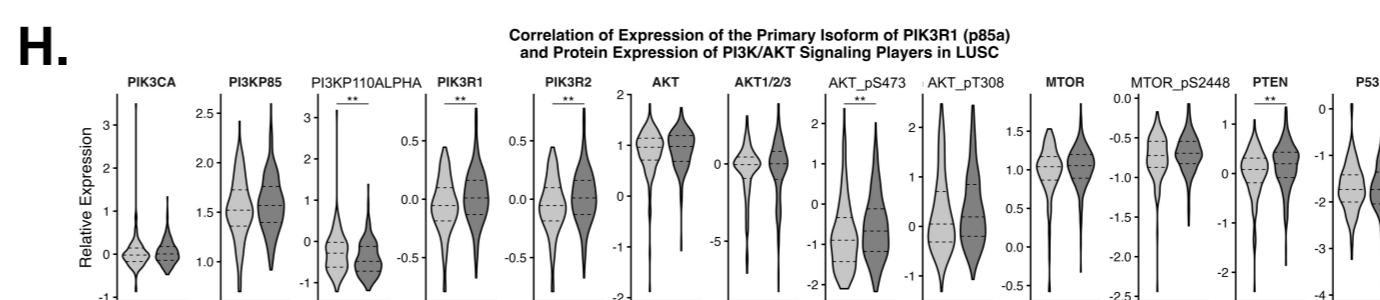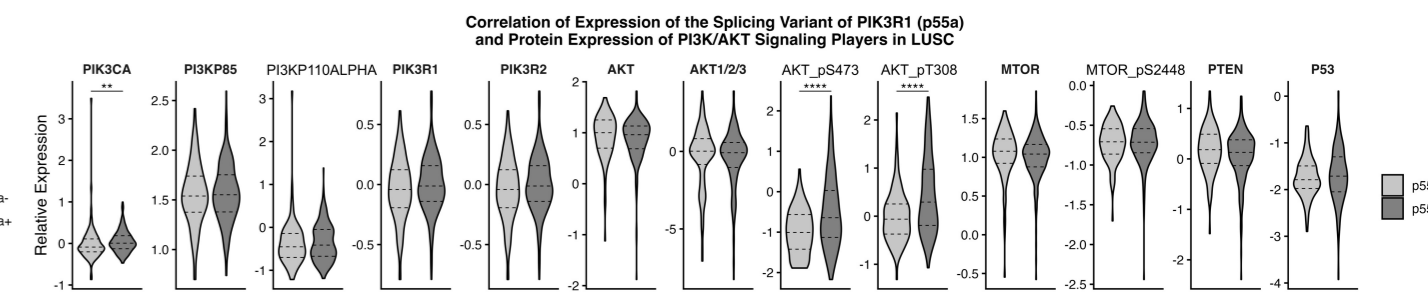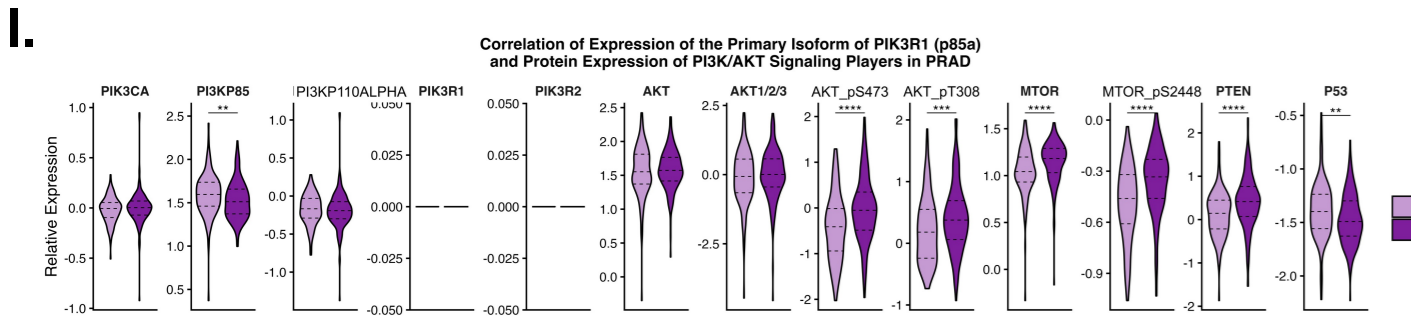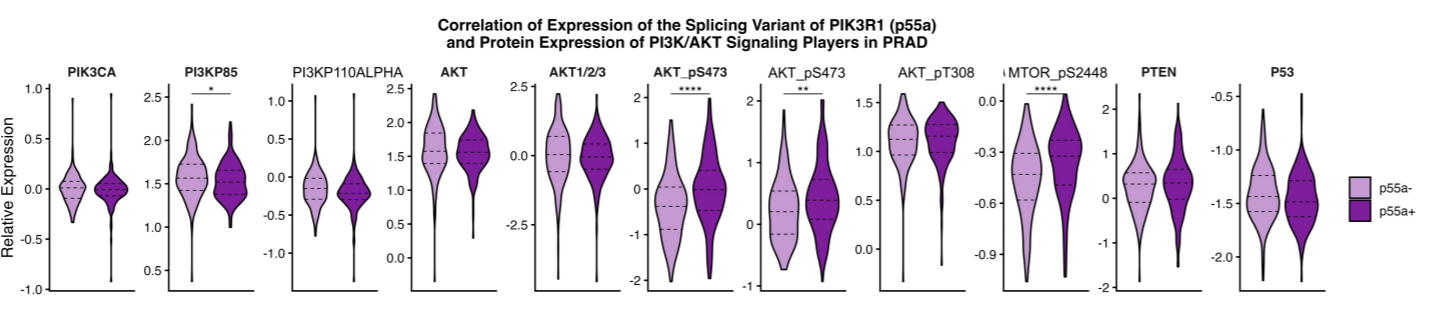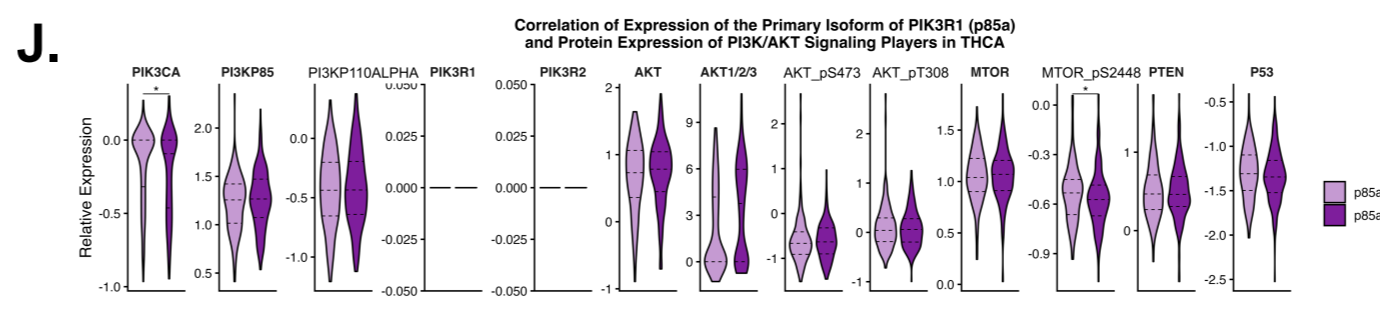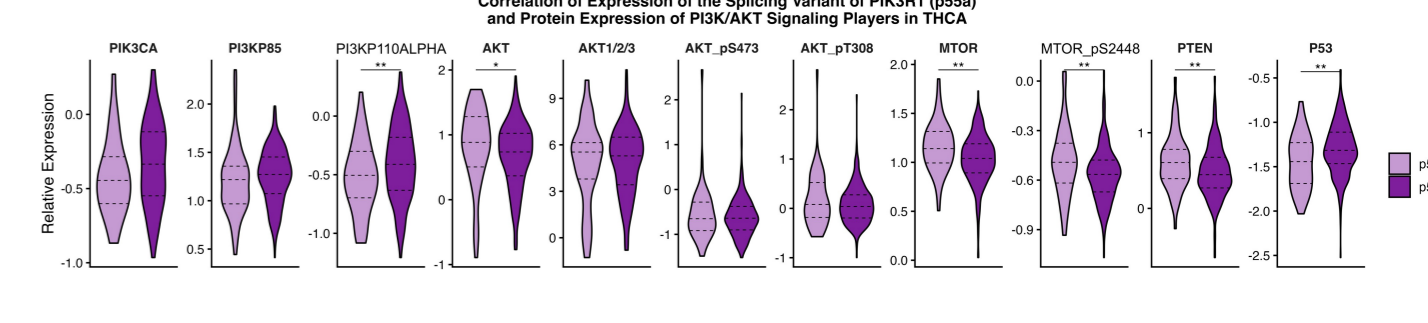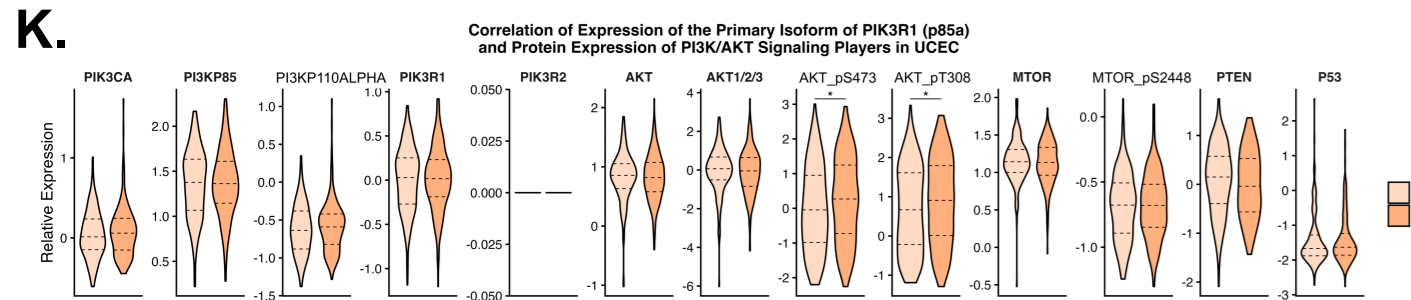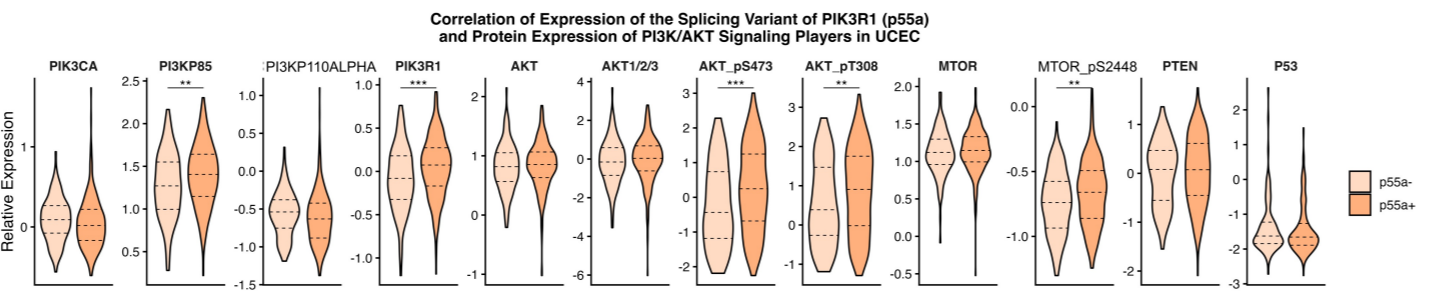

Supplement: Supplementary file 9 — Fig. S9. Correlation between the Expression Levels of the Primary Isoform of PIK3R1 (p85α) and the Splicing Variant of PIK3R1 (p55α) with Target Players of the PI3K/Akt Pathway, across the 11 TCGA cancer types including (A) BRCA, (B) HNSC (C) KICH, (D) KIRC, (E) KIRP, (F) LIHC, (G) LUAD, (H) LUSC, (I) PRAD, (J) THCA and (K) UCEC. [file MOL2-20-1299-s004.pdf]
