# Supplementary material for: Correlation of the differential expression of PIK3R1 and its spliced variant, p55α, in pan‐cancer
Source: Mol Oncol. 2026 Jan 20;20(5):1299–322. doi: 10.1002/1878-0261.70205 (PMC13155144; doi:10.1002/1878-0261.70205)
Supplement: Supplementary file 3 — Fig. S3. Correlation between the Expression Levels of the Primary Isoform of PIK3R1 (p85α) and Splicing Variant of PIK3R1 (p55α) with Overall Survival (OS) based on Racial Disparity, across (A) BRCA, (B) KICH, (C) KIRP, (D) LIHC, (E) PRAD, (F) THCA and (G) UCEC. [file MOL2-20-1299-s009.pdf]

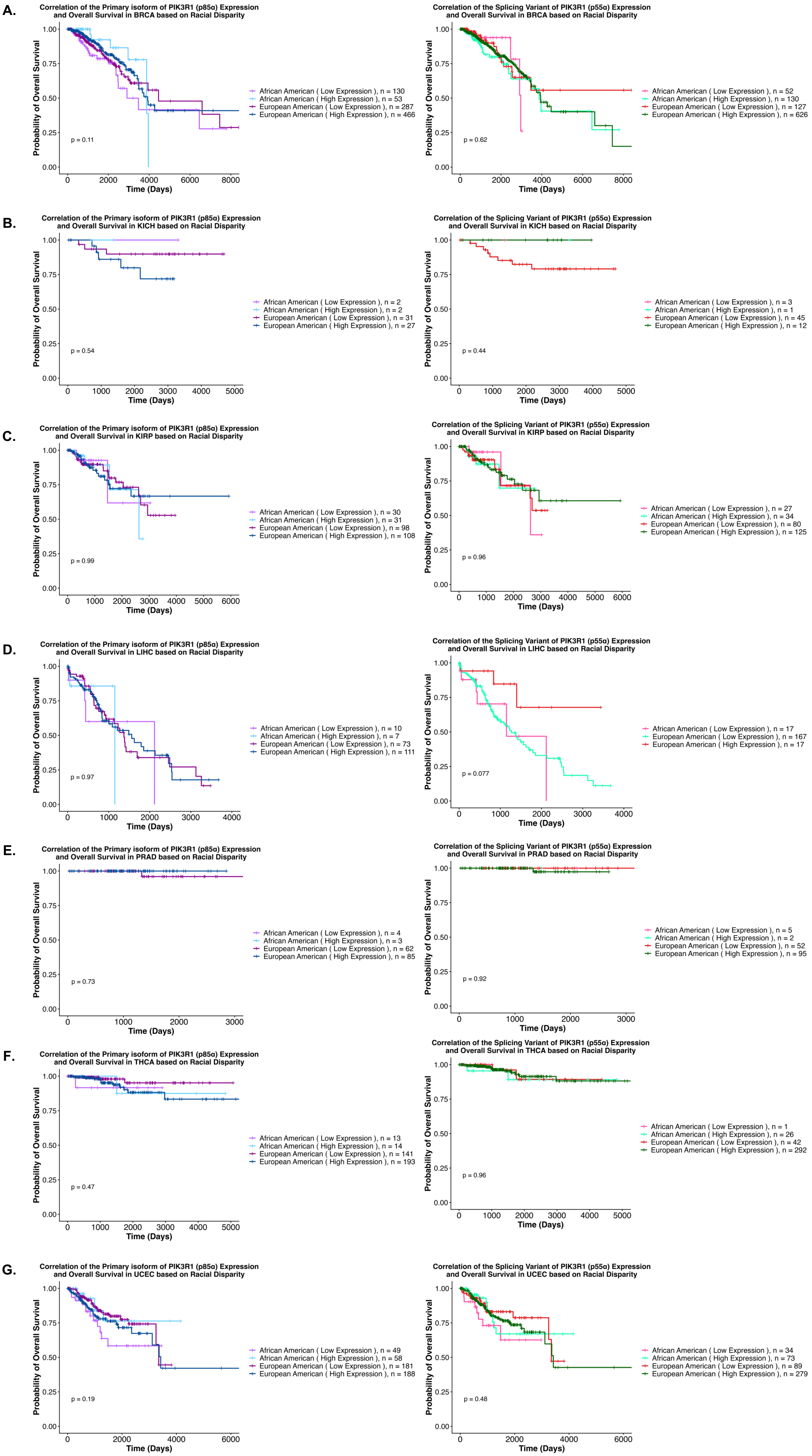
A.
Correlation of the Primary isoform of PIK3R1 (p85α) Expression and Overall Survival in BRCA based on Racial Disparity
Probability of Overall Survival
Time (Days)
p = 0.11
African American (Low Expression), n = 130
African American (High Expression), n = 53
European American (Low Expression), n = 287
European American (High Expression), n = 466
Correlation of the Splicing Variant of PIK3R1 (p55α) Expression and Overall Survival in BRCA based on Racial Disparity
p = 0.62
African American (Low Expression), n = 52
African American (High Expression), n = 130
European American (Low Expression), n = 127
European American (High Expression), n = 626
B.
Correlation of the Primary isoform of PIK3R1 (p85α) Expression and Overall Survival in KICH based on Racial Disparity
p = 0.54
African American (Low Expression), n = 2
African American (High Expression), n = 2
European American (Low Expression), n = 31
European American (High Expression), n = 27
Correlation of the Splicing Variant of PIK3R1 (p55α) Expression and Overall Survival in KICH based on Racial Disparity
p = 0.44
African American (Low Expression), n = 3
African American (High Expression), n = 1
European American (Low Expression), n = 45
European American (High Expression), n = 12
C.
Correlation of the Primary isoform of PIK3R1 (p85α) Expression and Overall Survival in KIRP based on Racial Disparity
p = 0.99
African American (Low Expression), n = 30
African American (High Expression), n = 31
European American (Low Expression), n = 98
European American (High Expression), n = 108
Correlation of the Splicing Variant of PIK3R1 (p55α) Expression and Overall Survival in KIRP based on Racial Disparity
p = 0.96
African American (Low Expression), n = 27
African American (High Expression), n = 34
European American (Low Expression), n = 80
European American (High Expression), n = 125
D.
Correlation of the Primary isoform of PIK3R1 (p85α) Expression and Overall Survival in LIHC based on Racial Disparity
p = 0.97
African American (Low Expression), n = 10
African American (High Expression), n = 7
European American (Low Expression), n = 73
European American (High Expression), n = 111
Correlation of the Splicing Variant of PIK3R1 (p55α) Expression and Overall Survival in LIHC based on Racial Disparity
p = 0.077
African American (Low Expression), n = 17
European American (Low Expression), n = 167
European American (High Expression), n = 17
E.
Correlation of the Primary isoform of PIK3R1 (p85α) Expression and Overall Survival in PRAD based on Racial Disparity
p = 0.73
African American (Low Expression), n = 4
African American (High Expression), n = 3
European American (Low Expression), n = 62
European American (High Expression), n = 85
Correlation of the Splicing Variant of PIK3R1 (p55α) Expression and Overall Survival in PRAD based on Racial Disparity
p = 0.92
African American (Low Expression), n = 5
African American (High Expression), n = 2
European American (Low Expression), n = 52
European American (High Expression), n = 95
F.
Correlation of the Primary isoform of PIK3R1 (p85α) Expression and Overall Survival in THCA based on Racial Disparity
p = 0.47
African American (Low Expression), n = 13
African American (High Expression), n = 14
European American (Low Expression), n = 141
European American (High Expression), n = 193
Correlation of the Splicing Variant of PIK3R1 (p55α) Expression and Overall Survival in THCA based on Racial Disparity
p = 0.96
African American (Low Expression), n = 1
African American (High Expression), n = 26
European American (Low Expression), n = 42
European American (High Expression), n = 292
G.
Correlation of the Primary isoform of PIK3R1 (p85α) Expression and Overall Survival in UCEC based on Racial Disparity
p = 0.19
African American (Low Expression), n = 49
African American (High Expression), n = 58
European American (Low Expression), n = 181
European American (High Expression), n = 188
Correlation of the Splicing Variant of PIK3R1 (p55α) Expression and Overall Survival in UCEC based on Racial Disparity
p = 0.48
African American (Low Expression), n = 34
African American (High Expression), n = 73
European American (Low Expression), n = 89
European American (High Expression), n = 279
