# Supplementary material for: Correlation of the differential expression of PIK3R1 and its spliced variant, p55α, in pan‐cancer
Source: Mol Oncol. 2026 Jan 20;20(5):1299–322. doi: 10.1002/1878-0261.70205 (PMC13155144; doi:10.1002/1878-0261.70205)
Supplement: Supplementary file 8 — Fig. S8. BaseScope Duplex Detection and Quantification of the Primary Isoform of PIK3R1 (p85α) and Splicing Variant of PIK3R1 (p55α). [file MOL2-20-1299-s007.pdf]

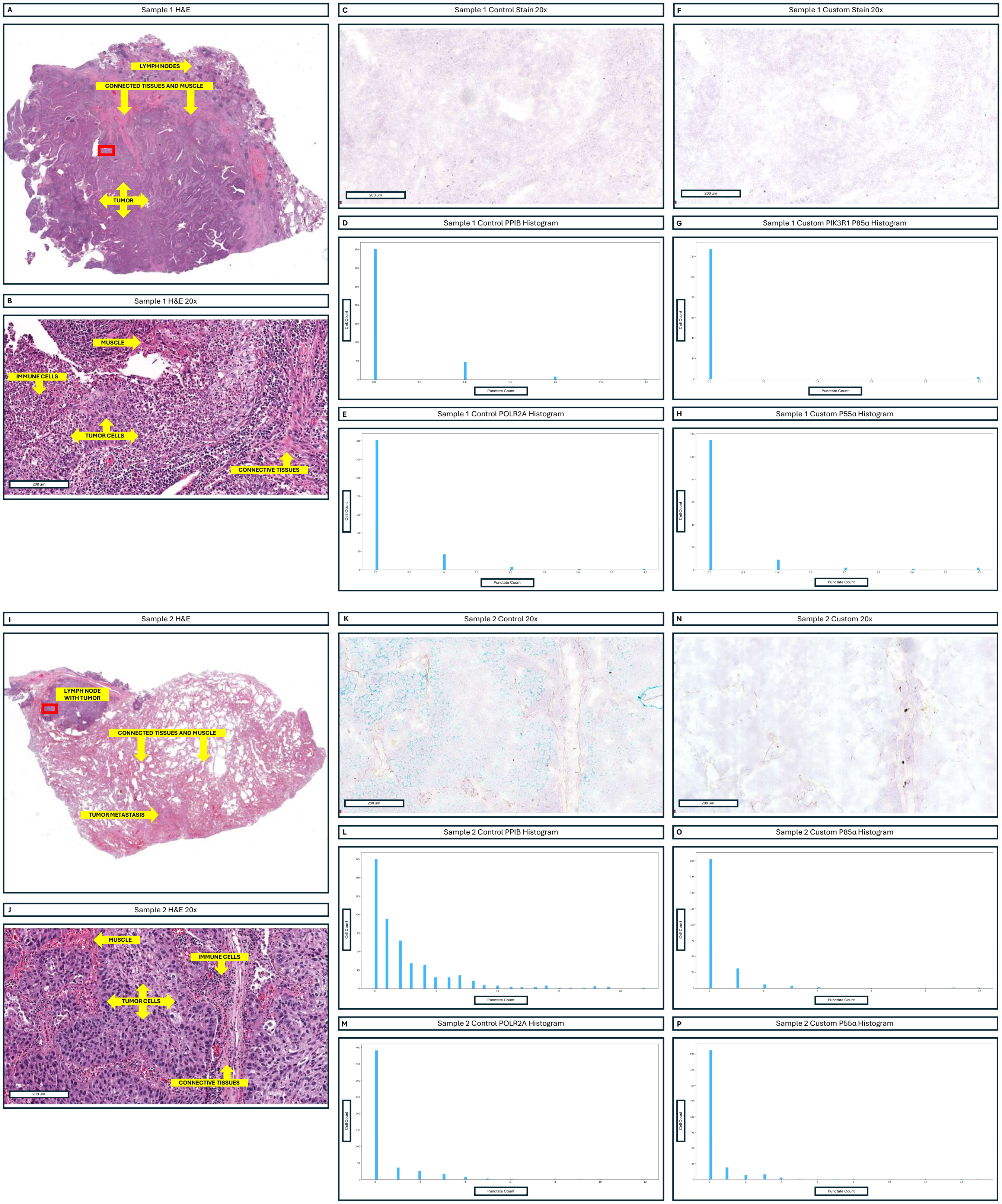

Figure [insert number]. BaseScope Duplex Detection and Quantification of PIK3R1 Isoforms
(A-B) Sample 1, probably good to include the type of cancer this is (we started with squamous lung, but I think we moved on to some adenocarcinoma samples later), not too much to say here
(C-H) Sample 1 control probes detect PPIB (green) and POLR2A (red). Histograms depict number of cells (y axis) with the number of punctate (x axis) indicated. Sample 1 custom probes detect long isoform (green) and short isoform (red)
(I-J) Sample 2, again, good to include type of cancer, perhaps some comments on the histology as well
(L-P) Same probes as earlier, same histograms as earlier
