# Supplementary material for: Correlation of the differential expression of PIK3R1 and its spliced variant, p55α, in pan‐cancer
Source: Mol Oncol. 2026 Jan 20;20(5):1299–322. doi: 10.1002/1878-0261.70205 (PMC13155144; doi:10.1002/1878-0261.70205)
Supplement: Supplementary file 10 — Table S1. List of cell lines used. The table includes the list of lung cancer cell lines used in the study. [file MOL2-20-1299-s006.docx]

**Supplementary Information**

**Supplemental Table 1. List of Lung Cancer Cell Lines Used**

| **Lung Cancer Cell Lines** | | | |
| --- | --- | --- | --- |
| **Cell Line** | **Research Resource Identifiers (RRIDs)** | **Type** | **In vitro validation** |
| Beas-2b | RRID:CVCL_0168 | Normal |  |
| Normal bronchial/tracheal epithelial (NHBE) | RRID:CVCL_1Q79 | Normal |  |
| A549 | RRID:CVCL_UJ49 | Tumor |  |
| NCI-H358 | RRID:CVCL_1559 | Tumor | YES |
| NCI-H1299 | RRID:CVCL_0060 | Tumor |  |
| NCI-H1703 | RRID:CVCL_1490 | Tumor |  |

**Supplementary Figures**

**Supplementary Figure 1 (A and B).** **Transcriptional Expression Analysis of the (A) Primary Isoform of *PIK3R1* (p85α) and (B) Its Splicing Variant (p55α).** The violin plot illustrates the transcriptional expression analysis of the primary isoform of *PIK3R1*, p85α, and its splicing variant, p55α, in the 32 cancer types analyzed between the normal and tumor tissue samples. The x-axis denotes the tissue type, while the y-axis indicates the relative expression level of the isoform. Statistical significance is denoted by asterisks: **p<0.05, **p<0.01, ***p<0.001, ****p<0.0001*. **N:** Normal tissue; **T:** Tumor tissue. **ACC:** Adrenocortical carcinoma; **BLCA:** Bladder Urothelial Carcinoma; **BRCA:** Breast invasive carcinoma; **CESC:** Cervical squamous cell carcinoma and endocervical adenocarcinoma; **CHOL:** Cholangiocarcinoma; **COAD:** Colon adenocarcinoma; **DLBC:** Lymphoid Neoplasm Diffuse Large B-cell Lymphoma; **ESCA:** Esophageal carcinoma; **GBM:** Glioblastoma multiforme; **HNSC:** Head and Neck squamous cell carcinoma; **KICH:** Kidney Chromophobe; **KIRC:** Kidney renal clear cell carcinoma; **KIRP:** Kidney renal papillary cell carcinoma; **LGG:** Brain Lower Grade Glioma; **LIHC:** Liver hepatocellular carcinoma; **LUAD:** Lung adenocarcinoma; **LUSC:** Lung squamous cell carcinoma; **MESO:** Mesothelioma; **OV:** Ovarian serous cystadenocarcinoma; **PAAD:** Pancreatic adenocarcinoma; **PCPG:** Pheochromocytoma and Paraganglioma; **PRAD:** Prostate adenocarcinoma; **READ:** Rectum adenocarcinoma; **SARC:** Sarcoma; **SKCM:** Skin Cutaneous Melanoma; **STAD:** Stomach adenocarcinoma; **TGCT:** Testicular Germ Cell Tumors; **THCA:** Thyroid carcinoma; **THYM:** Thymoma; **UCEC:** Uterine Corpus Endometrial Carcinoma; **UCS:** Uterine Carcinosarcoma; **UVM:** Uveal Melanoma.

**Supplementary Figure 2 (A-F). Correlation between the Expression Levels of the Primary Isoform of *PIK3R1* (p85α) and Splicing Variant of *PIK3R1* (p55α) with Overall Survival (OS).** The figure displays Kaplan-Meier survival curves illustrating the correlation between expression levels of the primary isoform of *PIK3R1*, p85α, and its splicing variant, p55α, with overall survival across the remaining 6 cancer types, **(A)** HNSC, **(B)** KIRP, **(C)** LIHC, **(D)** LUSC, (**E)** THCA and **(F)** UCEC. Each curve represents how the survival probability changes over time, with the x-axis denoting time in days and the y-axis representing the proportion of patients surviving. Curves are stratified based on the mean expression of the primary isoform of *PIK3R1*, p85α. On the other hand, for the spliced variant, p55α, curves are stratified based on p55α expression = '0' or > '0'. Statistical significance is determined using log-rank tests. The divergence or convergence of the curves indicates the potential impact of p85α or p55α expression on overall survival outcomes in the 6 cancer types. **HNSC:** Head and Neck squamous cell carcinoma; **KIRP:** Kidney renal papillary cell carcinoma; **LIHC:** Liver hepatocellular carcinoma; **LUSC:** Lung squamous cell carcinoma; **THCA:** Thyroid carcinoma; **UCEC:** Uterine Corpus Endometrial Carcinoma.

**Supplementary Figure 3 (A-G). Correlation between the Expression Levels of the Primary Isoform of *PIK3R1* (p85α) and Splicing Variant of *PIK3R1* (p55α) with Overall Survival (OS) based on Racial Disparity,** **across (A) BRCA, (B) KICH, (C) KIRP, (D) LIHC, (E) PRAD, (F) THCA and (G) UCEC.** The figure displays Kaplan-Meier survival curves illustrating the correlation between expression levels of the primary isoform of *PIK3R1*, p85α, and its splicing variant, p55α, with overall survival across the TCGA cancer types categorized by racial groups. Each curve represents how the survival probability changes over time, with the x-axis denoting time in days and the y-axis representing the proportion of patients surviving. Curves are stratified based on the mean expression of the primary isoform of *PIK3R1*, p85α. On the other hand, for the spliced variant, p55α, curves are stratified based on p55α expression = '0' or > '0'. The graph includes four distinct lines representing different patient groups, with each line depicting the probability of overall survival over time for the respective group. Statistical significance is determined using log-rank tests. The divergence or convergence of the curves indicates the potential impact of p85α or p55α expression on overall survival outcomes in the 7 cancer types based on racial disparity. **BRCA:** Breast invasive carcinoma; **KICH:** Kidney Chromophobe; **KIRP:** Kidney renal papillary cell carcinoma; **LIHC:** Liver hepatocellular carcinoma; **PRAD:** Prostate adenocarcinoma; **THCA:** Thyroid carcinoma; **UCEC:** Uterine Corpus Endometrial Carcinoma.

**Supplementary Figure 4 (A-G). Correlation between the Expression Levels of the Primary Isoform of *PIK3R1* (p85α) and Its Splicing Variant (p55α) with Progression-Free Interval (PFI).** The figure displays Kaplan-Meier survival curves illustrating the correlation between expression levels of the primary isoform of *PIK3R1*, p85α, and its splicing variant, p55α, with progression-free intervals across the remaining 7 cancer types, **(A)** KICH, **(B)** KIRP, **(C)** LIHC, **(D)** LUAD, (**E)** LUSC, **(F)** THCA and **(G)** UCEC. Each curve represents how the survival probability changes over time, with the x-axis denoting time in days and the y-axis representing the proportion of patients who have not experienced disease progression or recurrence. Curves are stratified based on the mean expression of the primary isoform of *PIK3R1*, p85α. On the other hand, for the spliced variant, p55α, curves are stratified based on p55α expression = '0' or > '0'. Statistical significance is determined using log-rank tests. The divergence or convergence of the curves indicates the potential impact of p85α or p55α expression on PFI outcomes in the 7 cancer types. **KICH:** Kidney Chromophobe; **KIRP:** Kidney renal papillary cell carcinoma; **LIHC:** Liver hepatocellular carcinoma; **LUAD:** Lung adenocarcinoma; **LUSC:** Lung squamous cell carcinoma; **THCA:** Thyroid carcinoma; **UCEC:** Uterine Corpus Endometrial Carcinoma.

**Supplementary Figure 5 (A-H). Correlation between the Expression Levels of the Primary Isoform of *PIK3R1* (p85α) and Its Splicing Variant (p55α) with Progression-Free Interval (PFI) based on Racial Disparity,** **across (A) KICH, (B) KIRP, (C) LIHC, (D) LUAD, (E) LUSC, (F) PRAD, (G) THCA and (H) UCEC.** The figure displays Kaplan-Meier survival curves illustrating the correlation between expression levels of the primary isoform of *PIK3R1*, p85α, and its splicing variant, p55α, with progression-free intervals across the remaining 8 cancer types categorized by racial groups. Each curve represents how the probability of recurrence changes over time, with the x-axis denoting time in days and the y-axis representing the proportion of patients who have not experienced disease progression or recurrence. Curves are stratified based on the mean expression of the primary isoform of *PIK3R1*, p85α. On the other hand, for the spliced variant, p55α, curves are stratified based on p55α expression = '0' or > '0'. The graph includes four distinct lines representing different patient groups, with each line depicting the probability of overall survival over time for the respective group. Statistical significance is determined using log-rank tests. The divergence or convergence of the curves indicates the potential impact of p85α or p55α expression on PFI outcomes in the 8 cancer types based on racial disparity. **KICH:** Kidney Chromophobe; **KIRP:** Kidney renal papillary cell carcinoma; **LIHC:** Liver hepatocellular carcinoma; **LUAD:** Lung adenocarcinoma; **LUSC:** Lung squamous cell carcinoma; **PRAD:** Prostate adenocarcinoma; **THCA:** Thyroid carcinoma; **UCEC:** Uterine Corpus Endometrial Carcinoma.

**Supplementary Figure 6 (A-C). Multivariate Cox Analysis identifying factors affecting Progression Free Interval.** The figure displays forrest plots for hazard ratios from the Cox proportional hazard multivariate models, illustrating the correlation between expression levels of the primary isoform of *PIK3R1*, p85α, and its splicing variant, p55α, with progression-free intervals across **(A)** BRCA, **(B)** HNSC, and **(C)** KIRC adjusting for age, sex, race, stage, smoking history, and alcohol history. The squares indicate hazard ratios and horizontal bars represent 95% confidence intervals; the vertical line at HR = 1 denotes no effect on survival. Statistical significance is denoted by asterisks: **p<0.05, **p<0.01, ***p<0.001, ****p<0.0001*. **BRCA:** Breast invasive carcinoma; **HNSC:** Head and Neck squamous cell carcinoma; **KIRC:** Kidney renal clear cell carcinoma.

**Supplementary Figure 7 (A and B). Relative expression of *PIK3R1* isoforms (p85α and p55α) after transient knockdown using siRNA specific to p85α and p55α for cell proliferation assay.** qRT-PCR was performed in H358 cells to analyze **(A)** p85α and **(B)** p55α relative mRNA levels normalized to the control gene (GAPDH). Relative expression values are expressed as fold changes; values less than one indicate downregulation (knockdown). Expression of p85α isoform was not affected by siRNA against p55α and vice-versa. Data represent the mean ± SD of three independent biological experiments, each performed in technical triplicate. The error bar represents the standard deviation. One-way ANOVA test was used to compare the target mRNA p85α and p55α expression between control siRNA, p85α and p55α siRNA.

**Supplementary Figure 8 (A-P). BaseScope Duplex Detection and Quantification of the Primary Isoform of *PIK3R1* (p85α) and Splicing Variant of *PIK3R1* (p55α). (A-H)** This figure illustrates the results of a BaseScope assay used to detect the expression of both the primary isoform of *PIK3R1*, p85α, and its splicing variant, p55α, in one LUSC tissue sample. The red square indicates the cancerous squamous cell region of that is magnified and shown in Figures B, C, and F. Whole-tissue images are shown at low magnification to illustrate overall tissue architecture (scale bar = 200 µm). Panels A–H correspond to the first tissue sample. **(A and B)** H&E-stained sections display tissue morphology. **(C)** Negative control using probes for housekeeping genes PP1B and POL2RA confirms minimal background. **(D and E)** Quantification of the negative control validates assay specificity. **(F)** BaseScope duplex staining reveals p85α (green dots) and p55α (red dots) expression at the single-cell level. **(G and H)** Bar graphs quantify the average number of p85α and p55α signals per cell across different tissue regions. Panels I–P follow the same structure for the second LUSC tissue sample. **(I and J)** H&E images of LUSC tissue sample show tissue architecture. The red square indicates the cancerous squamous cell region of that is magnified and shown in Figures J, K, and N. **(K)** Negative control demonstrates low background staining. **(L and M)** Quantification confirms specificity. **(N)** BaseScope results highlight distinct spatial expression patterns of p85α and p55α. **(O and P)** Bar graphs show regional quantification of each isoform. These images and data illustrate the differential expression and localization of *PIK3R1* isoforms, supporting their distinct roles in tumor biology.

**Supplementary Figure 9 (A-K). Correlation between the Expression Levels of the Primary Isoform of *PIK3R1* (p85α) and the Splicing Variant of *PIK3R1* (p55α) with Target Players of the PI3K/Akt Pathway, across the 11 TCGA cancer types including (A) BRCA, (B) HNSC (C) KICH, (D) KIRC, (E) KIRP, (F) LIHC, (G) LUAD, (H) LUSC, (I) PRAD, (J) THCA and (K) UCEC.** The figure displays violin plots illustrating the correlation between expression levels of the primary isoform of *PIK3R1*, p85α, and its splicing variant, p55α, with target players of the PI3K/Akt pathway (PIK3CA, p85α, p110a, PIK3R1, Akt, Akt1/2/3, Akt_pS473, Akt_pT308, mTOR, mTOR_pS448 and PTEN) across the 11 cancer types. The violin plots depict the distribution and density of the expression levels of the primary isoform of *PIK3R1* (p85α+) and its splicing variant (p55α+) in relation to various target proteins within the PI3K/Akt pathway. Each violin represents a different target protein, showing the spread of p85α and p55α expression levels across samples, with the width indicating the density of the data points at different expression levels. One-way ANOVA tests were performed to determine the significance of the differences in expression levels of each target protein based on the low and high expression of the primary isoform (p85α- and p85α+, respectively) as well as the splicing variant (p55α- and p55α+, respectively) of *PIK3R1*. Statistical significance is denoted by asterisks: **p<0.05, **p<0.01, ***p<0.001, ****p<0.0001*. Both panels provide a visual representation of the correlation between the *PIK3R1* isoforms and the target proteins, with the median and interquartile ranges marked within each violin. **BRCA:** Breast invasive carcinoma (BRCA); **HNSC:** Head and Neck squamous cell carcinoma; **KICH:** Kidney Chromophobe; **KIRC:** Kidney renal clear cell carcinoma; **KIRP:** Kidney renal papillary cell carcinoma; **LIHC:** Liver hepatocellular carcinoma; **LUAD:** Lung adenocarcinoma; **LUSC:** Lung squamous cell carcinoma; **PRAD:** Prostate adenocarcinoma; **THCA:** Thyroid carcinoma; **UCEC:** Uterine Corpus Endometrial Carcinoma.
